# Integration of transcriptome analysis with pathophysiological endpoints to evaluate cigarette smoke toxicity in an in vitro human airway tissue model

**DOI:** 10.1007/s00204-021-03008-0

**Published:** 2021-03-03

**Authors:** Rui Xiong, Yue Wu, Qiangen Wu, Levan Muskhelishvili, Kelly Davis, Priya Tripathi, Ying Chen, Tao Chen, Matthew Bryant, Hans Rosenfeldt, Sheila M. Healy, Xuefei Cao

**Affiliations:** 1 Division of Genetic and Molecular Toxicology, National Center for Toxicological Research, US Food and Drug Administration, Jefferson, AR 72079 USA; 2 Division of Bioinformatics and Biostatistics, National Center for Toxicological Research, US Food and Drug Administration, Jefferson, AR 72079 USA; 3 Division of Biochemical Toxicology, National Center for Toxicological Research, US Food and Drug Administration, Jefferson, AR 72079 USA; 4 Toxicologic Pathology Associates, Jefferson, AR 72079 USA; 5 Office of Scientific Coordination, National Center for Toxicological Research, US Food and Drug Administration, Jefferson, AR 72079 USA; 6 Division of Nonclinical Science, Center for Tobacco Products, US Food and Drug Administration, Silver Spring, Maryland 20993 USA

**Keywords:** Cigarette smoke (CS), Air–liquid-interface (ALI) airway tissue model, Pathophysiologically relevant endpoints, Transcriptomic profiling, Chronic obstructive pulmonary disease (COPD)

## Abstract

Exposure to cigarette smoke (CS) is a known risk factor in the pathogenesis of smoking-caused diseases, such as chronic obstructive pulmonary diseases (COPD) and lung cancer. To assess the effects of CS on the function and phenotype of airway epithelial cells, we developed a novel repeated treatment protocol and comprehensively evaluated the progression of key molecular, functional, and structural abnormalities induced by CS in a human in vitro air–liquid-interface (ALI) airway tissue model. Cultures were exposed to CS (diluted with 0.5 L/min, 1.0 L/min, and 4.0 L/min clean air) generated from smoking five 3R4F University of Kentucky reference cigarettes under the International Organization for Standardization (ISO) machine smoking regimen, every other day for 4 weeks (3 days per week, 40 min/day). By integrating the transcriptomics-based approach with the in vitro pathophysiological measurements, we demonstrated CS-mediated effects on oxidative stress, pro-inflammatory cytokines and matrix metalloproteinases (MMPs), ciliary function, expression and secretion of mucins, and squamous cell differentiation that are highly consistent with abnormalities observed in airways of smokers. Enrichment analysis on the transcriptomic profiles of the ALI cultures revealed key molecular pathways, such as xenobiotic metabolism, oxidative stress, and inflammatory responses that were perturbed in response to CS exposure. These responses, in turn, may trigger aberrant tissue remodeling, eventually leading to the onset of respiratory diseases. Furthermore, changes of a panel of genes known to be disturbed in smokers with COPD were successfully reproduced in the ALI cultures exposed to CS. In summary, findings from this study suggest that such an integrative approach may be a useful tool for identifying genes and adverse cellular events caused by inhaled toxicants, like CS.

**Supplementary Information:**

The online version contains supplementary material available at 10.1007/s00204-021-03008-0.

## Introduction

Cigarette smoke (CS) is a complex mixture of over 7000 chemicals (USDHHS 2014). Of these chemicals, 93 have been established by FDA as Harmful or Potentially Harmful Constituents (HPHCs) in tobacco products and tobacco smoke, being identified as carcinogens, respiratory toxicants, cardiovascular toxicants, reproductive or developmental toxicants, and addictive agents (FDA 2012). Cigarette smoking adversely affects nearly every tissue in the body, particularly the respiratory system, contributing to the initiation and progression of both malignant and nonmalignant lung diseases, such as lung cancer, asthma, and chronic obstructive pulmonary disease (COPD) (USDHHS 2014). Smoking is considered one of the most common preventable causes of morbidity and mortality in the United States (CDC 2008).

Long-term CS exposure is the primary risk factor for developing the chronic obstructive pulmonary disease (COPD). It accounts for 85–90% of the incidence and approximately 80% of COPD-related death (Pauwels et al. 2001; USDHHS 2010; USDHHS 2014). The underlying pathogenesis of COPD is multifaceted. In general, it is characterized by irreversible obstruction of airflow associated with persistent airway inflammation, tissue destruction, and disturbance of mucin homeostasis (Laniado-Laborin 2009). The increased oxidant burden from CS induces oxidative stress, which, in turn, initiates inflammatory responses (Brody and Spira 2006). Reactive oxygen/nitrogen species released by the epithelial and immune cells result in further oxidative damage, leading to perpetual oxidative stress and chronic inflammatory reactions in the lung. These molecular initiating events eventually cause the modification of tissue structure and function that are characteristic of COPD (Kirkham and Barnes 2013).

Although extensive knowledge about CS health effects has been gained from decades of tobacco research, toxicity evaluation of CS remains challenging due to the complexity of tobacco products, variations in smoking behavior, and lack of testing standards (Brownawell 2007; St. Hilaire 2007). Given the limitations of animal models, there is a degree of uncertainty in using animal data for evaluating and predicting human responses (Bracken 2009). New alternative methods (NAMs), therefore, have been devised to address key challenges and facilitate evaluating the hazard posed by CS in humans. NAMs have proven invaluable in prioritizing further research and generating mechanistic insight to inform adverse outcome pathway analysis (Clippinger et al. 2018).

The epithelial lining of the airways interacts directly with inhaled substances. The airway lining functions as the first line of defense against inhaled toxicants and pathogens, not only by providing a physical barrier but also through mucociliary clearance mechanisms and immunological defense functions (Tam et al. 2011). Considering its critical role in responses to toxicant exposures, elucidating the cellular and tissue changes in airway epithelium could provide useful information on the toxicity and disease outcomes of inhaled toxicants.

Primary human bronchial epithelial cells can be differentiated into organotypic pseudo-stratified epithelium at the air–liquid interface (ALI). ALI airway models have a complex tissue structure that retains many of the cell types and tissue architecture found in airway epithelium in vivo. These unique properties, along with their ability to remain functional for months, provide a means for generating data in vitro using extended CS treatment regimens. Our laboratory has tested a variety of CS solutions and constituents to evaluate the suitability of human airway ALI models for toxicity assessments and, as a result of these studies, developed a panel of respiratory disease-relevant toxicity endpoints (Cao et al. 2017, 2018; Xiong et al. 2018, 2019). In these studies, we observed a general concordance of abnormalities induced by CS constituents and fractions, such as impaired cilia activity, dysfunction in mucin secretion, and aberrant tissue remodeling (e.g., squamous differentiation) between in vitro and in vivo responses.

**Fig. 1:**
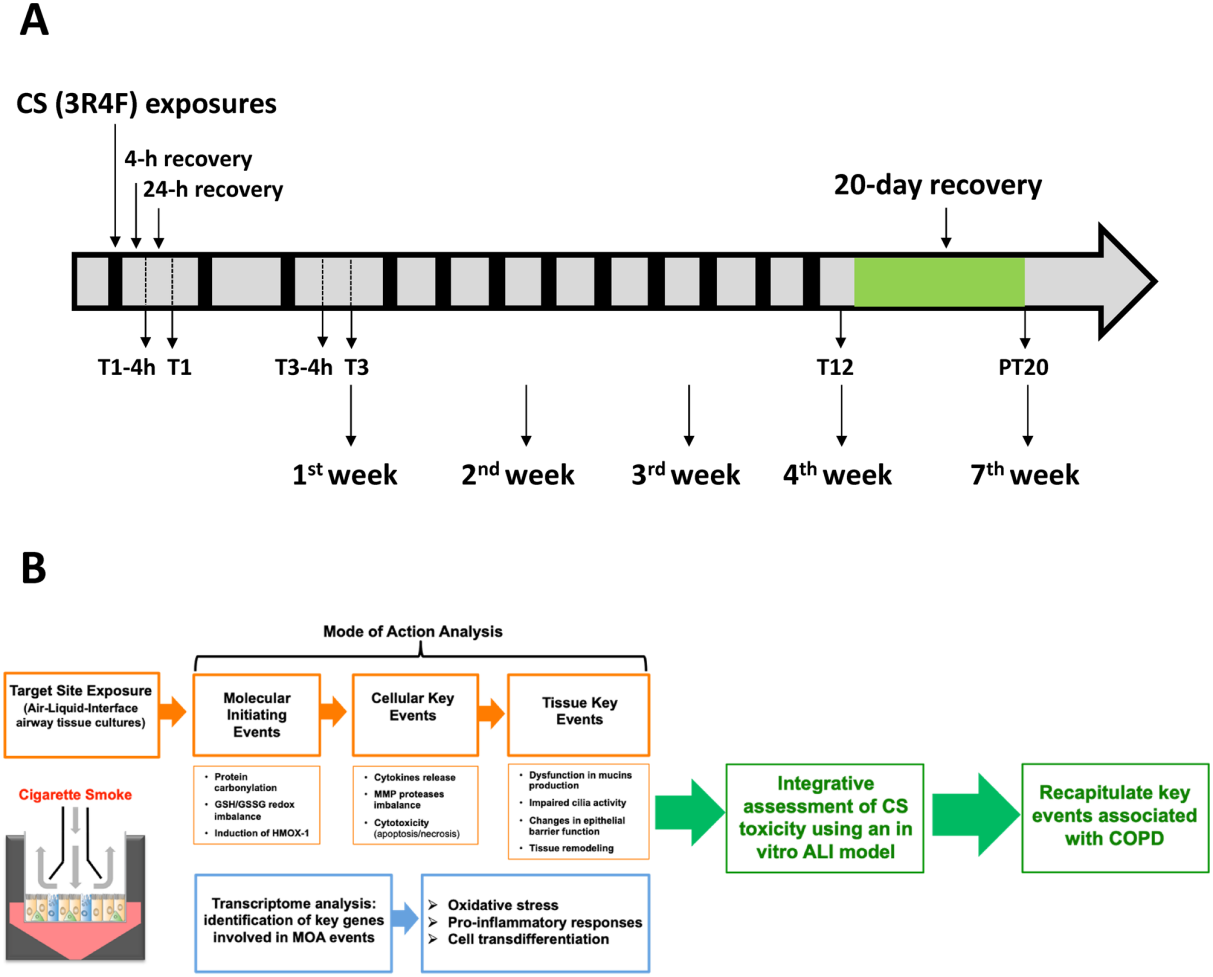
Schematic representation of a 1-month CS exposure and proposed integrative approach for assessing CS toxicity in the ALI airway tissue models. Cultures were exposed to CS aerosols generated by smoking 5 3R4F reference cigarettes (**a**). Smoke-treated cultures were collected 24 h after 1 and 12 exposures (T1 and T12) as well as after a 20-day recovery (PT20) for toxicity analyses as illustrated in the MOA of CS toxicity (**b**). Note that early sampling times, including a 4- and 24-h recovery after 1 and 3 exposures (T1-4h, T1, T3-4h, and T3), were included in the RNA-seq time-course study to assess the dynamic molecular changes induced by CS exposure (color figure online)

In this study, we evaluated the pathophysiological responses, such as cilia beating, mucin homeostasis, epithelial integrity, and tissue morphology, modulated by CS in human airway ALI cultures. A novel repeated treatment regimen (40 min per day, 3 times per week, for a total of 4 weeks, illustrated in Fig. 1a) was employed to establish the association of subacute responses with the key events depicted in the proposed mode of action (MOA) of CS as well as to distinguish acute stress responses from adaptive/modified responses (Fig. 1b). Transcriptome analysis was integrated with the pathophysiological evaluations at several key timepoints to elucidate the molecular basis of the functional and structural changes induced by CS. Furthermore, the reversibility of the molecular and tissue responses was assessed to understand the persistence of the toxic responses.

## Materials and methods

### Human air–liquid-interface airway tissue models

Human ALI airway tissue models were established as described previously (Xiong et al. 2018). Normal human bronchial epithelial (NHBE) cells (MatTek, Ashland, MA) were expanded in PneumaCult™-Ex Medium (STEMCELL Technologies, Vancouver, Canada). Cells were seeded at a density of 4.0 × 10^4^ cells onto 24-well Transwell^®^ culture inserts and grown in the Expansion Medium until they reached 100% confluence. Medium from the apical side was then removed and cultures were fed from the basolateral side with PneumaCult™-ALI Maintenance Medium (STEMCELL Technologies) for 4 weeks.

### Cigarette smoke generation

3R4F reference cigarettes (University of Kentucky, KY) were conditioned in an environmental chamber (Caron, Marietta, OH) for a minimum of 48 h according to ISO 3402:1999 (22 ± 1 ℃, relative humidity of 60 ± 2%). Whole CS was freshly generated using a Vitrocell^®^ VC10 S-Type (VC10-S) Rotary Smoking Robot equipped with a 10-port smoke head and 2 independent syringe pumps (Waldkirch, Germany). Performance of the smoking robot was assessed by verifying the air velocity (200 ± 30 mm/s), system leakage, and puff volume (35.0 ± 0.3 mL) according to ISO 3308:2012 before each exposure. Whole CS was generated by smoking 5 3R4F reference cigarettes according to ISO 4387:2000 (35 mL per puff over 2 s every 60 s, to a defined number of 8 puffs per cigarette).

### Whole smoke exposure

Whole CS was diluted to three concentrations by introducing dilution air (ultra-zero synthetic air, 85% nitrogen and 15% oxygen, nexAir, Memphis, TN) at 4.0, 1.0, and 0.5 L/min (hereafter referred to as the low, mid, and high CS concentration) using a Vitrocell^®^ Dilution System. Diluted smoke then was drawn into a Vitrocell^®^ 12/12 Exposure System by vacuum at a flow rate of 2.0 mL/min. Two Exposure Systems were connected linearly to increase the number of replicate cultures exposed to each concentration. A Quartz Crystal Microbalance (QCM) was connected at the end of each dilution line to record CS particle deposition every 2 s throughout the smoke exposure. ALI airway cultures were gently washed twice with DPBS before being placed into positions in the exposure modules that contained sufficient Maintenance Medium. The cultures were then exposed to diluted CS at the ALI for 40 min (5 cigarettes per exposure, 8 min per cigarette), 3 exposures every week on Monday, Wednesday, and Friday, for 4 consecutive weeks. After each exposure, ALI cultures were washed with DPBS from the basolateral side only and maintained in a new 24-well plate containing fresh Maintenance Medium (400 µL per well) until the next exposure. During the recovery phase, ALI cultures were washed from both the apical and basolateral sides every other day and maintained in Maintenance Medium (500 µL per well) for 20 days (Fig. 1a). Clean air-exposed cultures were included as concurrent vehicle controls.

### Mass spectrometry analysis of nicotine concentration in the exposure medium

Nicotine quantification was adapted from a method described previously (Haswell et al. 2017) with modifications on sample preparation procedures. The medium collected at the end of each exposure was mixed with acetonitrile containing 50 ng/mL of the internal nicotine-d_4_ standard at a ratio of 1:4 (v/v), vortexed, and centrifuged at 22,000 × *g* for 5 min. Supernatant (2 µL) was injected onto a Waters ACQUITY UPLC System coupled with a Waters ACQUITY QDa Mass Detector (Milford, MA). Nicotine and nicotine-d_4_ were eluted on an ACQUITY UPLC HSS T3 column (2.1 mm × 50 mm, 1.8 μm) at 40˚C with mobile phases consisting of 10 mM ammonium acetate (A) and acetonitrile (B), both containing 0.1% formic acid, at a flow rate of 0.5 mL/min. Elution started with 7% Solvent B for 0.1 min, followed by a linear gradient of 7–40% Solvent B over 0.9 min, returning to 7% Solvent B in 0.2 min, and maintained at 7% Solvent B for 0.8 min to re-equilibrate the column. The eluate was analyzed using mass spectrometry with an electrospray ion source operating in the positive ion mode (ESI +) using single ion recording (SIR). The monitored (M + H)^+^ ions were m/z 163.05 for nicotine and m/z 167.10 for nicotine-d_4_. Nicotine was quantified using a calibration curve ranging from 0.78 to 1000 ng nicotine/mL with Waters Empower 3 software. Concentrations of nicotine were expressed as ng nicotine/mL exposure medium.

### Lactate dehydrogenase activity assay

The cytotoxicity of CS was monitored 24 h after 1 (T1) and 12 intermittent exposures (T12), as well as after a 20-day recovery period (PT20) using a Lactate Dehydrogenase (LDH) Activity Assay kit (Roche, Indianapolis, IN). Basolateral media was collected at T1, T12, and PT20 and assayed for LDH release. Equal volumes of the basolateral medium and freshly prepared Reaction Mixture were mixed and allowed to react for 15 min at room temperature in the dark. Reactions were terminated by the Stop Solution. Absorbance at 490 nm was measured using a Synergy H4 microplate reader (BioTek, Winooski, VT).

### Protein carbonyl ELISA

The intracellular level of protein carbonyls was measured immediately after CS exposure (acute) and at T1, T12, as well as PT20. Protein carbonyls were quantified using a Protein Carbonyl ELISA kit and by following the manufacturer’s instructions (CellBio Labs, San Diego, CA) as described previously (Xiong et al. 2018). Briefly, ELISA plates were coated with 100 µL standards or 1 µg cell lysate diluted in 100 µL Dulbecco’s phosphate-buffered saline (DPBS) at 4 ℃ overnight. Carbonyl groups were derivatized with an equal volume of 0.04 mg/mL 2,4-dinitrophenylhydrazine (DNPH) for 45 min at room temperature. Derivatized proteins were detected using an anti-DNP antibody and a HRP-conjugated secondary antibody, followed by color development using the kit-provided 3,3′,5,5′-Tetramethylbenzidine (TMB) substrate. The absorbance was measured at 450 nm using a BioTek Synergy H4 microplate reader. Reduced bovine serum albumin (BSA) standard was used as the background control.

### Intracellular GSH and GSSG levels

Modulation of GSH homeostasis in pulmonary tissues is associated with the development and progression of inflammatory lung diseases, including COPD (Rahman and MacNee 1999). As an additional endpoint assessing CS-induced oxidative stress, the intracellular levels of GSH and GSSG were quantified at the same time points as those for protein carbonyl quantification (i.e., acute, T1, T12, and PT20) using a modified liquid chromatography-mass spectrometry (LC–MS) method (Xiong et al. 2018).

### Immunoblotting

Expression of key protein markers was measured at T1, T12, and PT20 using immunoblotting as described previously (Xiong et al. 2018). Briefly, proteins were denatured in a loading buffer containing 50 mM dithiothreitol (DTT), separated on a NuPage^®^ Novex^®^ 4–12% Bis–Tris gradient gel (Life Technologies, Carlsbad, CA), and transferred onto a nitrocellulose membrane (LI-COR, Lincoln, NE). Proteins were detected by incubating first with primary antibodies (rabbit anti-HMOX-1 antibody, Cell Signaling, Danvers, MA; mouse anti-acetylated α-tubulin, Sigma-Aldrich, St. Louis, MO; mouse anti-CK6, Santa Cruz, Dallas, TX; or mouse anti-involucrin, Thermo Fisher Scientific, Waltham, MA) and then IRDye-conjugated secondary antibodies (LI-COR). Images were taken using the LI-COR Odyssey^®^ CLx Imaging System and densitometry performed using the LI-COR Image Studio software.

### Bio-plex human cytokine and matrix metalloproteinase (MMP) assays

The proinflammatory properties of CS contribute to the initiation and progression of inflammatory respiratory diseases, including asthma and COPD (Strzelak et al. 2018). The inflammatory response triggered by CS has been correlated with MMP-mediated extracellular matrix remodeling in the lungs of smokers (Perlstein and Lee 2006). To assess these inflammatory responses to subacute exposures to CS, we quantified the secretion of select cytokines, chemokines, and MMPs into the basolateral medium at T12 and PT20. The Bio-plex Pro Human Cytokine 27-plex assay kit and a Human MMP 9-plex assay kit (Bio-Rad, Hercules, CA) were used for quantifying the secretion of select cytokines and MMPs into the basolateral medium. Briefly, basolateral media were incubated first with fluorescent magnetic beads by shaking vigorously at 850 ± 50 rpm for 1 h at room temperature, followed by incubation with the Detection Antibodies. The protein-antibody conjugates were visualized with streptavidin-PE. Fluorescence of the beads was measured and quantified using a MAGPIX system (Luminex, Austin, TX).

### Mucin ELISA

Maintaining mucin homeostasis is vital for respiratory health (Ridley and Thornton 2018). The effects of CS on the intracellular expression and secretion of 3 airway mucin proteins, i.e., MUC5AC, MUC5B, and club cell secretory protein (CCSP) were examined at T12 and PT20 using an ELISA method. Apical washes were collected into 200 µL DPBS. The disulfide bonds in the mucins were disrupted by adding DTT to a final concentration of 0.025 mM. Cell debris was removed by centrifugation at 600 × *g* for 10 min at 4 °C. Mucin samples (50 µL apical washes for mucin secretion analysis and 3 µg whole cell lysates for mucin expression analysis) were loaded onto a NUNC-Immuno^®^ MaxiSorp™ ELISA plate (Thermo Fisher Scientific); the plate was dried in a 37 °C incubator overnight. Detection of the mucin proteins was conducted as described previously (Xiong et al. 2018).

### Cilia beating frequency

Cilia beating frequency (CBF) in response to subacute CS exposure was measured at T12 and PT20. Cultures were equilibrated to 30 °C on a heated stage before measuring CBF. The beating of ciliated cells was captured using a high-speed camera (Ammons Engineering, Mount Morris, MI) affixed to a Leica DMI4000B microscope (Buffalo Grove, IL). Four fields devoid of mucus clumps were randomly selected and videotaped for each culture. CBF was analyzed using Sisson-Ammons Video Analysis (SAVA) software (Ammons Engineering).

### Histology and immunohistochemistry

Morphological changes caused by subacute exposure to CS were assessed at T12 and PT20. Culture inserts were washed with DPBS and fixed in 10% neutral buffered formalin for 48 h at 4 °C. The insert membranes then were excised from the plastic support, routinely processed, embedded in paraffin, sectioned to approx. 4–5-µm thickness, mounted on positively charged glass slides, deparaffinized with xylene, and rehydrated with ethanol solutions of decreasing concentrations. One set of the tissue sections was stained with hematoxylin and eosin (H&E) using a Leica Autostainer (Buffalo Grove, IL), dehydrated in ethanol, cleared with xylene, and finally mounted with Permount™ Mounting Medium (Fisher Scientific, Pittsburg, PA).

Goblet cells were detected by staining a separate set of tissue sections for mucopolysaccharides with periodic acid-Schiff (PAS). Briefly, the slides were deparaffinized with xylene, rehydrated in ethanol solutions of decreasing concentration to distilled water, placed in 0.5% periodic acid for 5 min, rinsed in distilled water, stained with Schiff’s reagent for 30 min, and rinsed in running tap water for 10 min. The slides next were counterstained with Gill’s Hematoxylin III for 15 s, rinsed in 3 changes of distilled water, placed in Scott’s Bluing Reagent for 1 min, dipped 3 times in distilled water, dehydrated in ethanol, cleared with xylene, and mounted with Permount™ Mounting Medium. Goblet cell density was calculated by counting the PAS-positive cells over a 5-mm length in the center of the tissue section and expressed as PAS-positive cells per mm.

The effect of CS on cell turnover was evaluated on tissue sections immunohistochemically stained with anti-cleaved caspase-3, anti-p63, and anti-Ki-67 antibodies for identifying apoptotic bodies, basal cells, and proliferating cells, respectively, at T12 and PT20. Squamous differentiation was assessed using anti-involucrin antibody. Expression of involucrin is up-regulated in human airway epithelium that has undergone squamous differentiation, a common pathological lesion observed in habitual smokers (Rigden et al. 2016). The tissue sections mounted on slides were placed in an antigen retrieval solution (0.01 M citrate buffer, pH 6.0) for 15 min in a microwave oven set at 110 °C and 800 W. For detection of involucrin, the antigen was retrieved in 1 mg/mL trypsin for 5 min at 37 °C. Tissue sections then were incubated in 3% hydrogen peroxide for 10 min to inactivate the endogenous peroxidases. Nonspecific staining was blocked in 0.5% casein (for Ki67 and cleaved caspase-3 staining) or 10% rat serum (for p63 and involucrin staining) for 20 min. The primary antibodies (diluted 1:100 in DPBS) were applied to the sections and incubated in a moisturized chamber for 1 h at room temperature. Following a 5-min wash in DPBS, biotinylated goat anti-rabbit or rat anti-mouse secondary antibodies (diluted 1:200 in DPBS, Jackson ImmunoResearch Laboratories, West Grove, PA) were applied to the tissue sections and incubated for 30 min at room temperature. The slides then were washed and the sections incubated in ExtrAvidin peroxidase (Sigma-Aldrich). Positively stained cells were visualized in 3′-diaminobenzidine (Sigma-Aldrich) for 5 min at room temperature. Sections were counter-stained with hematoxylin, dehydrated, cleared in xylene, and mounted with Permount™ Mounting Medium. The number of Ki67- or p63-positive nuclei as well as cleaved caspase-3-positive apoptotic bodies were counted over a 5-mm length in the center of the tissue section and expressed as Ki67-, p63-, or cleaved caspase 3-positive cells per mm. All counts were done under the light microscope (BX40, Olympus, Japan).

### Trans-epithelial electrical resistance

Tissue permeability was assessed at T12 and PT20 by measuring changes in trans-epithelial electrical resistance (TEER) using an EVOM2 epithelial volt-ohmmeter and a chopstick electrode (World Precision Instruments, Sarasota, FL). Prior to the TEER measurement, ALI cultures were briefly washed twice with DPBS from the apical side to remove the excess mucus. Cultures were then equilibrated to room temperature in fresh Maintenance Medium (400 µL per well). DPBS (200 µL) was added to the apical chamber of the culture inserts. Measurements on each culture were made at 3 positions that were 120° apart and the average was used for data analysis.

### RNA-Seq analysis

Aberrant tissue remodeling caused by CS exposure, such as squamous differentiation and metaplasia, is resulted from a multi-step process initiated by a cascade of molecular events, including responses to oxidative stress, induction of proinflammatory mediators as well as abnormal cell differentiation (Jetten 1989). Herein, a high-throughput RNA-Seq was used to analyze changes in the transcriptome profiles induced by CS at multiple time points. As changes in gene expression occur earlier than those in protein expression, samples were also collected 4 h after 1 (T1-4h) and 3 (T3-4h) exposures as well as 24 h after 3 exposures (T3), in addition to the T1, T12, and PT20 time points. Total RNA (1 μg), extracted using a RNeasy Mini Kit (Qiagen, Gaithersburg, MD), was sent to Novogene (Sacramento, CA) for RNA-Seq library preparation and sequencing. Reads in FASTQ files were first aligned to the human genome using the STAR aligner (v2.5). HTSeq (v0.6.1) was used to count the read numbers mapped for each gene. Differential expression analysis between the treatment and control groups (three biological replicates per group) was performed using the DESeq2 R package (2_1.6.3). The resulting *p*-values were adjusted using Benjamini–Hochberg approach for controlling False Discovery Rate (FDR). Genes with an adjusted *p*-value (*p*_adj_) of less than 0.05 were considered differentially expressed. The cluster Profiler R package was then used to test the enrichment of differentially expressed genes into different pathways as defined by Gene Ontology (GO, www.geneontology.org), KEGG (http://www.kegg.jp/), and Human Disease Ontology (DO, http://www.disease-ontology.org). GO, KEGG, and DO terms with *p*_adj_ < 0.05 were considered significantly enriched by differentially expressed genes.

### Statistical analysis

**Fig. 2:**
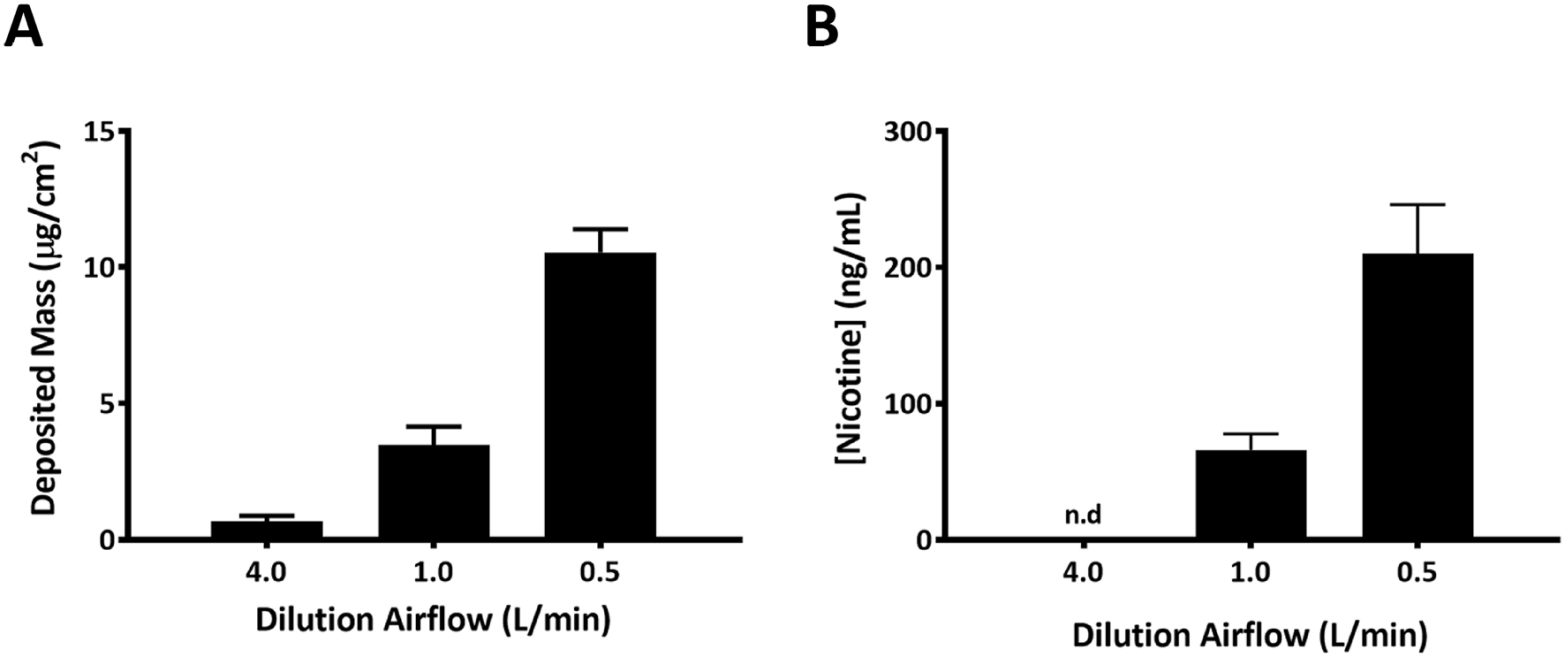
Smoke dosimetry. Dosimetry of CS was evaluated using QCMs connected in-line to the exposure module (**a**) and by quantifying the concentrations of nicotine in the exposure medium collected at the end of each smoke exposure (**b**). Nicotine concentration in the low concentration group was not detected. Each experiment consists of 12 exposures. The data expressed as means ± SD are the average of the 12 independent smoke exposures (*n* = 12 for the QCM data and *n* = 48 for nicotine concentrations). n.d: not detected

All experiments were conducted at least three times and the data (except the dosimetry data in Fig. 2) are presented as means ± standard error of the mean (SEM). Statistical analysis was performed using GraphPad Prism (La Jolla, CA). Unless otherwise indicated, *p* value < 0.05 was considered statistically significant compared to the respective clean air-exposed control by one-way ANOVA followed by Dunnett’s multiple comparison test.

## Results

### Smoke dosimetry assessment

To ensure the repeatability of CS generation, two methods were used concurrently with cell culture exposures to evaluate smoke dosimetry. First, particle deposition was recorded for each CS dilution using in-line QCMs (Fig. 2a). A linear increase in the deposition of smoke particles was observed. Deposited particle mass on the QCMs for the low, mid, and high CS concentrations were recorded as 0.681 ± 29.37% relative standard deviation (RSD), 3.478 ± 19.24%RSD, and 10.54 ± 8.11%RSD µg/cm^2^ for each 40-min exposure, which is equivalent to a particle mass of 0.14–2.11 µg/cm^2^ per 3R4F cigarette (particle mass/cigarette = average of QCM/5 cigarettes). Quantification of nicotine concentrations in the exposure medium was conducted for each CS dilution as an additional method to assess the repeatability of CS delivery (Fig. 2b). The nicotine concentration in the exposure medium collected from the low CS concentration group was below the detection limit of the analytical method. A proportional increase in nicotine concentration was observed at the mid and high CS concentrations. The nicotine concentrations (ng/mL) in the mid and high concentration groups are 65.8 ± 18.4%RSD and 210.1 ± 17.2%RSD, respectively. As can be inferred from the standard deviations of these measurements, both CS dosimetry evaluation conducted in parallel with cell exposures demonstrated consistent and repeatable generation and delivery of smoke aerosols throughout the study.

### Cytotoxicity of CS

**Fig. 3:**
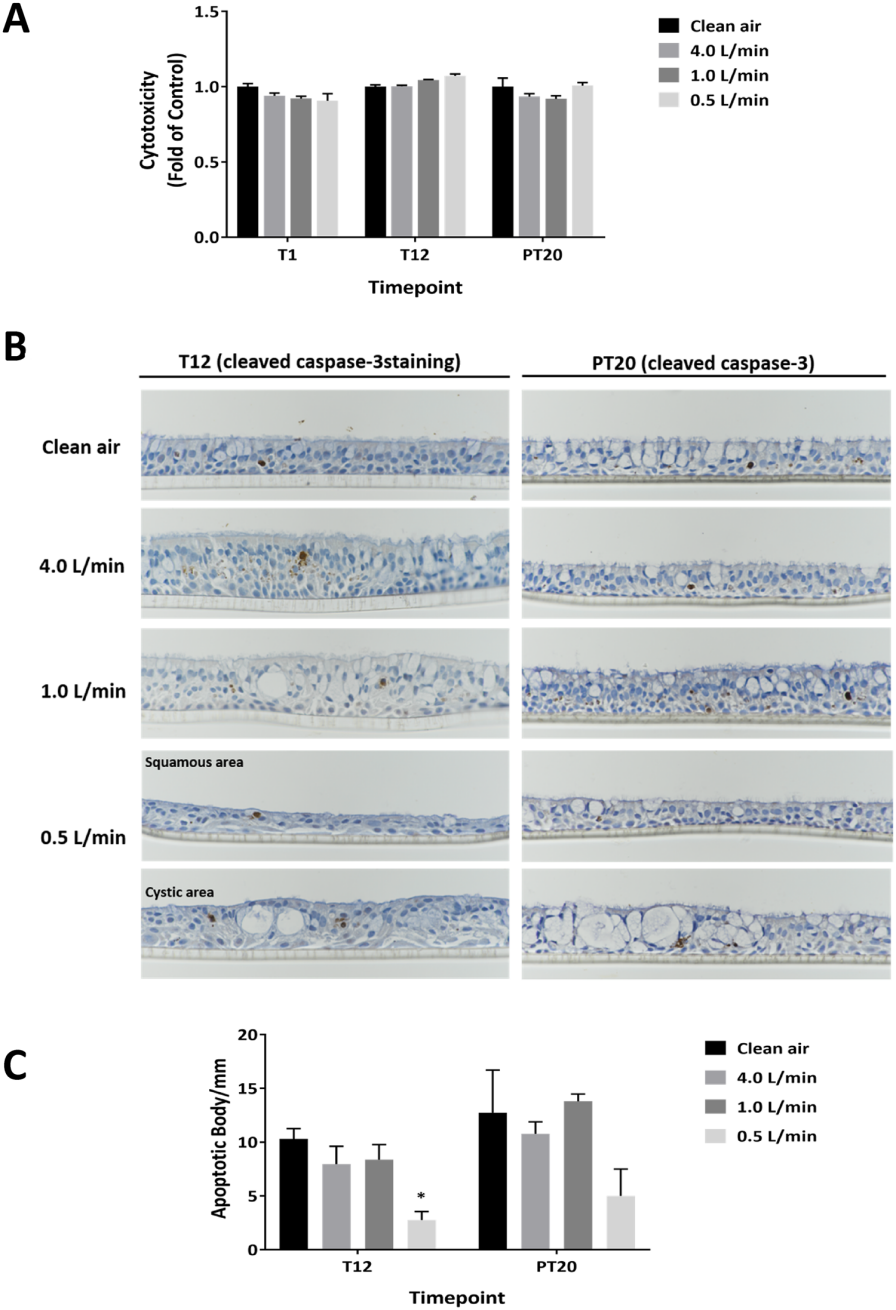
Cytotoxicity of CS in the ALI cultures. Cytotoxicity associated with CS was evaluated using the LDH activity assay at T1, T12 as well as PT20 (**a**). Apoptotic index was evaluated by staining tissue sections with a cleaved caspase-3 antibody at T12 and PT20 (**b**). Images were taken using 40 × magnification. The density of cleaved caspase-3-positive cells was calculated (**c**). Data (*n* = 6 for the LDH assay; *n* = 3 for the immunohistochemical staining) are expressed as means ± SEM. *p < 0.05 was considered statistically significant compared to the corresponding clean air-exposed control by one-way ANOVA using Dunnet’s multiple comparison test (color figure online)

Minimal cytotoxicity was observed at all CS concentrations throughout the exposure and recovery phases (Fig. 3a). The percentage of apoptotic bodies (ABs) was less than 15% in all groups at both time points (Fig. 3b, T12 and PT20). Examination of the tissue sections, however, indicated that exposure to the high concentration of CS caused heterogeneous structural changes to the tissue sections (see below). In areas of the epithelium where cilia and goblet cells were absent or where squamous differentiation was present, ABs were also absent (Fig. 3b, T12). A 20-day recovery period partially restored the structure of the damaged tissues (Fig. 3b, PT20). Quantitative analysis of the immunohistochemically-stained sections revealed a consistent decrease in ABs in cultures exposed to the high concentration of CS (Fig. 3c, T12). After a 20-day recovery period, inhibition of AB formation in these cultures persisted, although to a lesser extent and the decrease was not statistically significant (Fig. 3c, PT20).

### Induction of oxidative stress by CS

**Fig. 4:**
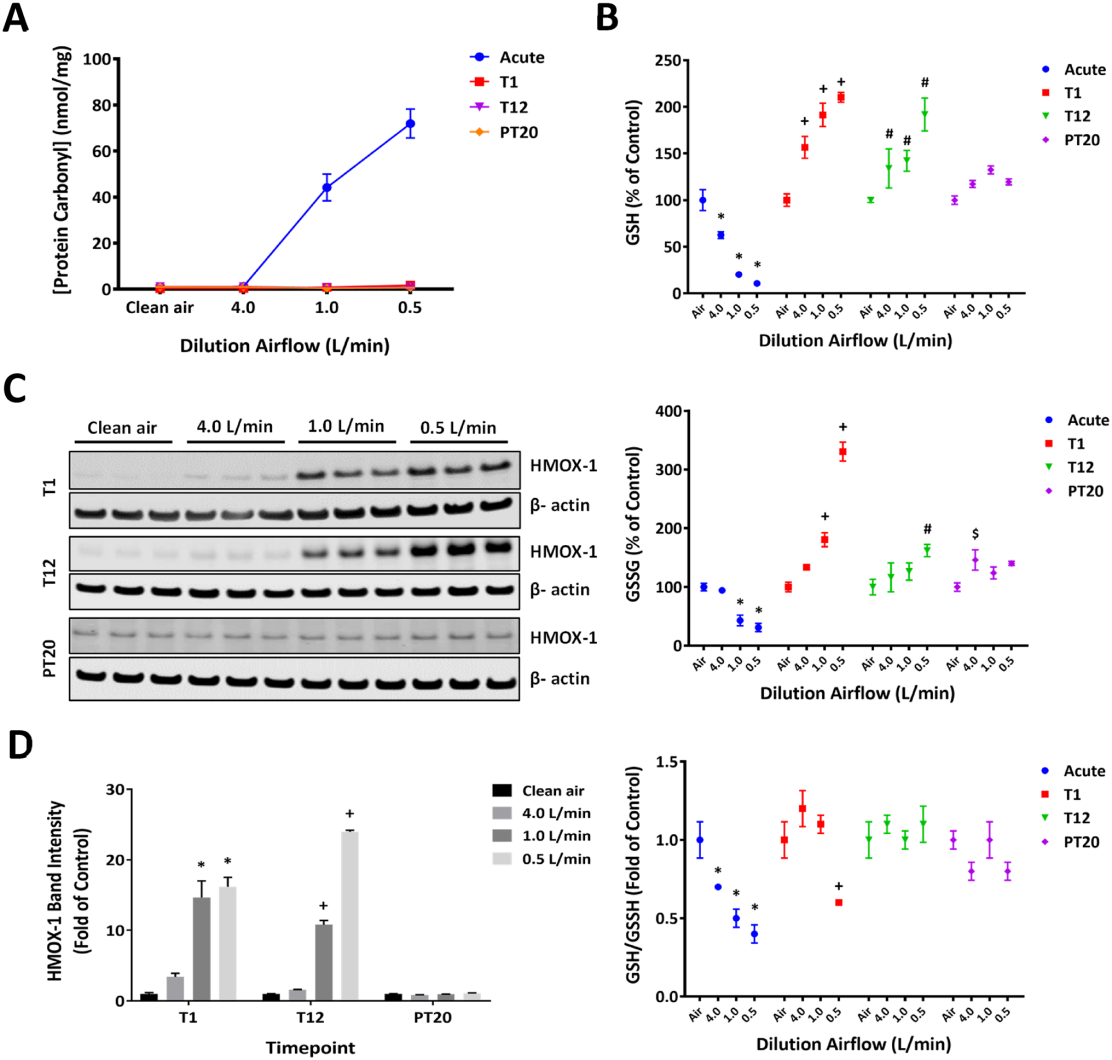
Induction of oxidative stress by CS. Time-dependent induction of protein oxidation by CS exposure was assessed by quantifying the intracellular levels of protein carbonyls at multiple time points (**a**). Disturbance of the glutathione homeostasis by CS exposure was assessed by quantifying the intracellular levels of GSH and GSSG (**b**). Induction of HMOX-1 protein expression was evaluated at T1, T12, and PT20 by immunoblotting (**c**). β-Actin was used to normalize the loading of the proteins. Band intensity of HMOX-1 was quantified by densitometry (**d**). Data (*n* = 3) are expressed as means ± SEM. *^,+, #,$^*p* < 0.05 was considered statistically significant compared to the respective clean air-exposed controls by one-way ANOVA using Dunnet’s multiple comparison test (color figure online)

Induction of oxidative stress by CS exposure was assessed using three methods that probe different mechanisms, including protein oxidation (Dalle-Donne et al. 2003), GSH/GSSG redox balance (Jones 2002), and induction of heme oxygenase-1, HMOX-1 (Choi and Alam 1996). Formation of protein carbonyls was induced immediately following the first exposure (Fig. 4a, acute). Their levels returned to the baseline at T1 and T12 and throughout the recovery phase (Fig. 4a, T1, T12, and PT20). Given the temporary nature of protein oxidation induction, the formation of protein carbonyls was independent of the number of exposures.

Significant concentration-dependent decreases in the intracellular levels of GSH and GSSG were observed immediately following the first CS exposure (Fig. 4b, top and middle panels, acute). As the CS-mediated depletion of GSH occurred to a greater extent than the formation of GSSG, the ratio of GSH/GSSG also was decreased (Fig. 4b, bottom panel, acute). Both GSH and GSSG levels were markedly increased in a concentration-dependent manner 24 h after the first exposure (Fig. 4b, top and middle panels, T1). Furthermore, the level of GSSG in the high CS concentration group was significantly greater than that of GSH, resulting in a decrease in the GSH/GSSG ratio (Fig. 4b, bottom panel, T1). Following 12 CS exposures, the intracellular levels of GSH and GSSG remained elevated but were at comparable levels (Fig. 4b, top and middle panels, T12). The GSH/GSSG ratio, therefore, appeared not to be affected by CS exposure at this time point (Fig. 4b, bottom panel, T12). The levels of GSH and GSSG as well as the GSH/GSSG ratio returned to the baseline after a 20-day recovery (Fig. 4b, PT20).

Expression of HMOX-1 was increased in a concentration-dependent manner, with the high concentration of CS causing more than 16-fold increase in its expression (Fig. 4c, d, T1). Its levels remained elevated after 12 CS exposures (Fig. 4c, d, T12), but returned to baseline levels after a 20-day recovery (Fig. 4c, d, PT20).

### Modulation of cytokine and MMP secretions by CS

**Fig. 5:**
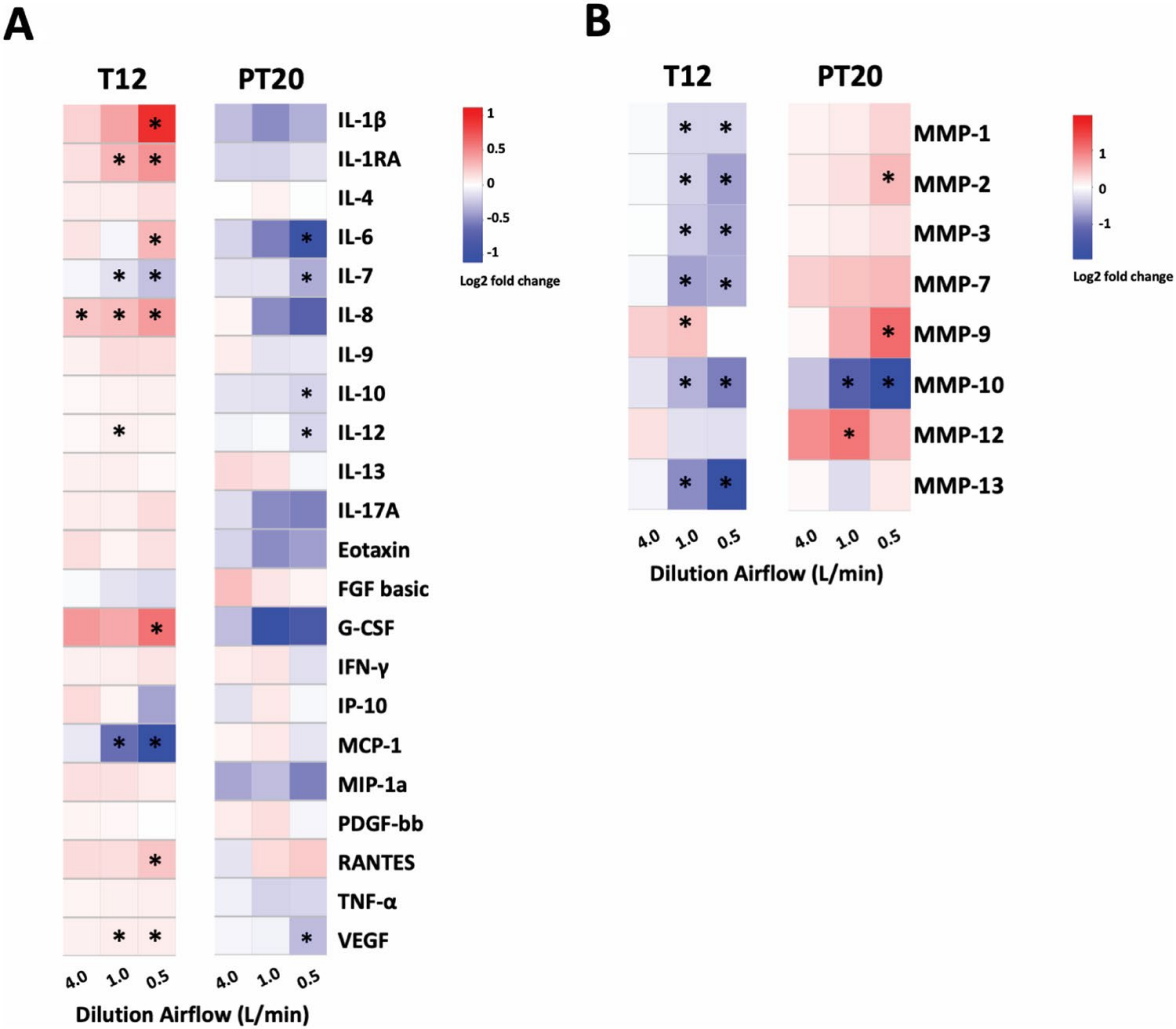
CS-mediated modulation of cytokine and MMP secretion. Concentration-dependent secretion of select proinflammatory cytokines (**a**) and MMPs (**b**) induced by CS exposures were quantified using the Bio-plex assay kits at T12 and PT20. The heatmaps were produced using gplots package of open-source R software. Data (*n* = 4) are expressed as means ± SEM. *p < 0.05 was considered statistically significant compared to the respective clean air-exposed controls by one-way ANOVA using Dunnet’s multiple comparison test (color figure online)

Release of IL-1β, IL-1RA (interleukin-1 receptor antagonist), IL-6, IL-8, G-CSF, and RANTES (i.e., CCL5) was significantly increased 24 h after 12 exposures (Fig. 5a, T12). Among the cytokines stimulated by CS exposure, the secretion of IL-1β was increased approx. 6.5 folds at the high CS concentration and in a concentration-dependent manner. Secretion of IL-1RA, IL-8, and G-CSF also increased in a concentration-dependent manner, although to a lesser extent. Release of both IL-6 and RANTES was increased to approx. 1.6-fold in the high CS concentration group. On the contrary, secretion of two proinflammatory cytokines, IL-7 and monocyte chemoattractant protein-1 (MCP-1) were concentration-dependently inhibited by CS exposure, with MCP-1 being inhibited by as much as 90%. After a 20-day recovery, the secretion of all cytokines, except IL-6, returned to levels comparable to that of the clean air-exposed vehicle control (Fig. 5a, PT20). Unlike the stimulative effect on IL-6 secretion observed during the treatment phase, secretion of IL-6 was decreased at the end of the recovery by as much as 60%. Secretion of IL-7 remained decreased in the treated groups at PT20, although the inhibition was lessened.

In the current study, the release of 8 MMPs into the basolateral medium was evaluated at the T12 and PT20 sampling times. Secretion of all MMPs, except MMP-9 and -12, were inhibited after 12 intermittent exposures (Fig. 5b, T12). Among these MMPs, the release of MMP-10 and -13 was significantly inhibited by approximately 60% and 90%, respectively, in the high CS concentration group. Decreases in the secretions of other MMPs, including MMP-1, -2, -3, and -7, were less, with the extent of inhibition ranging from 30 to 50%. Secretion of MMP-9, on the other hand, was increased approx. 1.6-fold by the mid CS concentration. At the end of the recovery phase, secretion of MMP-1, -3, -7, and -13, returned to levels comparable to the clean air-exposed controls (Fig. 5b, PT20). The effects of CS on the release of MMP-10 (inhibitory) and MMP-12 (stimulatory) persisted after a 20-day recovery (Fig. 5b). In contrast to the inhibitory effect on MMP-2 release observed at T12, CS exposure increased MMP-2 secretion by approx. 1.3-fold, which persisted through the 20-day recovery. The release of MMP-12 was increased by approx. 1.7-fold by the mid CS concentration after a 20-day recovery, despite that no change was observed at the T12 sampling time.

### Disturbance of mucin homeostasis by CS

**Fig. 6:**
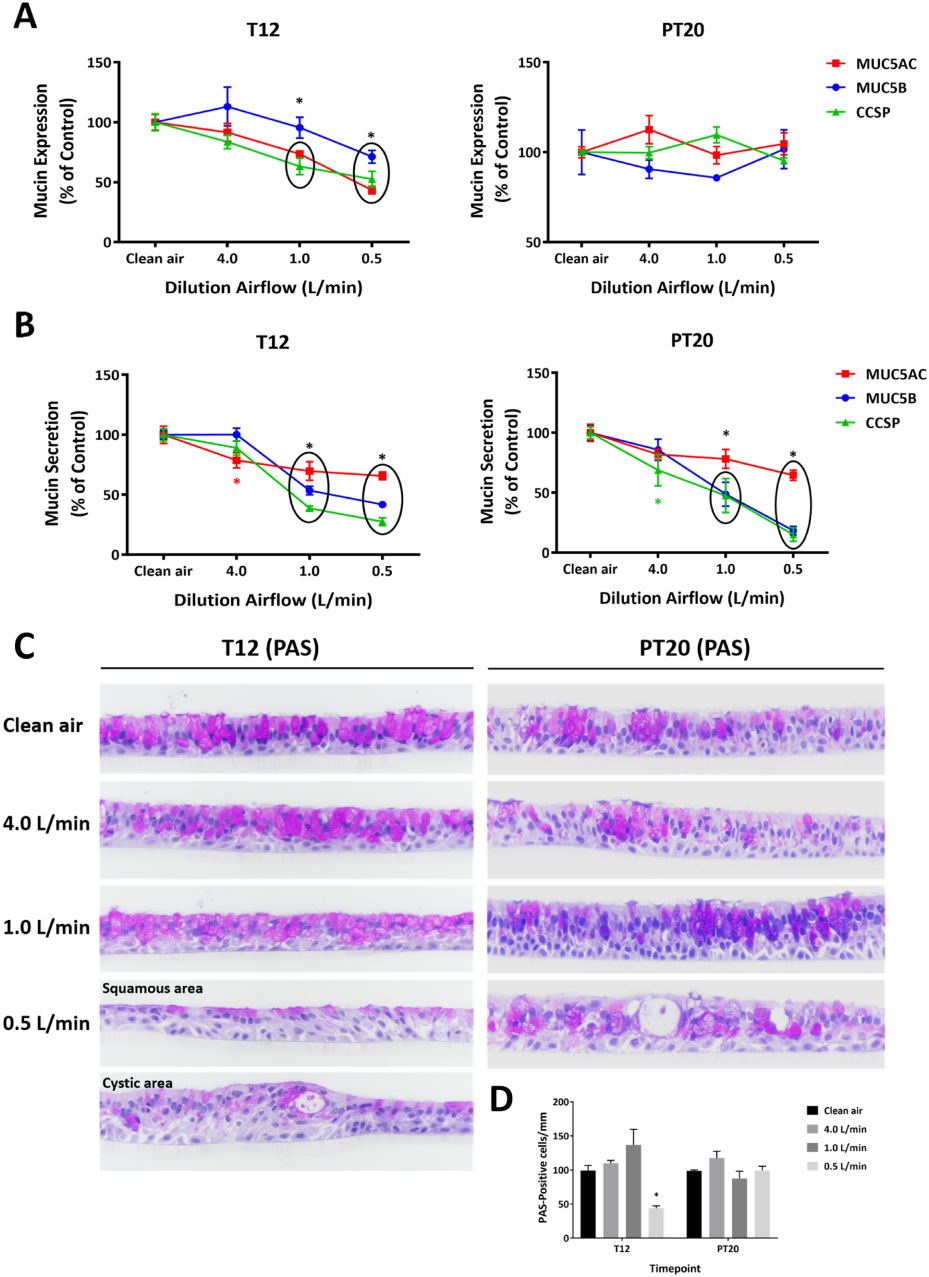
Disturbance of mucin homeostasis by CS. Expression (**a**) and secretion (**b**) of MUC5AC, MUC5B, and CCSP were evaluated at T12 and PT20. Morphological changes of goblet cells were assessed by PAS staining at the same time points (**c**). Pictures were taken using 40 × magnification. Density of goblet cells in a 5-mm length in the center of each tissue section was calculated as the PAS-positive cells per mm of tissue section (**d**). Data (*n* = 3 for mucin expression and *n* = 6 for mucin secretion) are expressed as means ± SEM. *p < 0.05 was considered statistically significant compared to the respective clean air-exposed controls by one-way ANOVA using Dunnet's multiple comparison test (color figure online)

Expression of mucin proteins, including MUC5AC, MUC5B, and CCSP, was down-regulated in a concentration-dependent manner after 12 exposures (Fig. 6a, T12) and returned to baseline levels at the PT20 sampling time (Fig. 6a, PT20). Secretion of mucins, on the other hand, was suppressed by CS exposures at both sampling times (Fig. 6b, T12 versus PT20).

In addition to its effects on mucins, loss of ciliated cells and goblet cells was observed in cultures exposed to the high concentration of CS (Fig. 6c, T12). The density of goblet cells was decreased by approx. 50% at the T12 sampling time (Fig. 6d, T12). Although goblet cell density in the CS-exposed cultures returned to a level comparable to that of the clean air-exposed control following a 20-day recovery (Fig. 6d, PT20), the structure of goblet cells was only partially restored. Histological examination indicated that goblet cells in the treated cultures appeared smaller compared to those in the clean air-exposed controls (Fig. 6c, PT20).

### Impaired ciliary structures and functions by CS

**Fig. 7:**
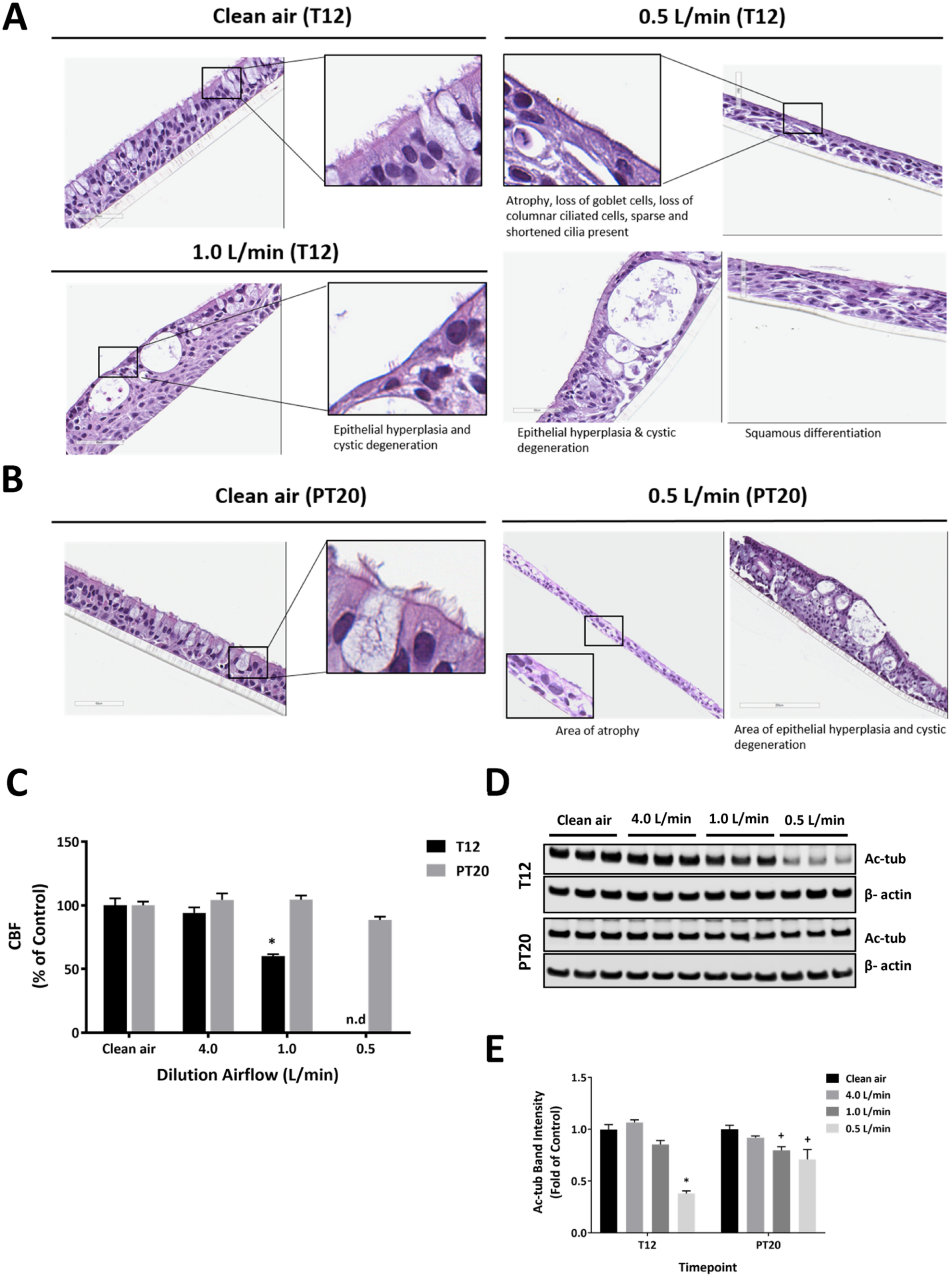
Impaired ciliary structure and function by CS. Loss of ciliated cells was assessed by H&E staining at T12 (**a**) and PT20 (**b**). Images were taken using 40 × magnification. Representative areas were magnified and shown in the insets. CBF was measured at T12 and PT20 (**c**). Protein expression of acetylated α-tubulin was evaluated at the same time points by immunoblotting (**d**). The intensity of the bands was quantified by densitometry and normalized to the β-actin loading control (**e**). Data (*n* = 3) are expressed as means ± SEM. *^,+^*p* < .05 was considered statistically significant compared to the respective clean air-exposed controls by one-way ANOVA using Dunnet’s multiple comparison test. n.d: not detected (color figure online)

Repeated exposure to the mid and high concentrations of CS resulted in a number of ciliary abnormalities at the T12 sampling time (Fig. 7a, T12). Cilia abnormalities consisted of decreased number of cilia, cilia of reduced length and/or absence of cilia in low cuboidal apical epithelial cells compared to the ciliated columnar epithelial cells of the vehicle controls. Areas with ciliary abnormalities coincided with areas that had cystic degeneration (one or more round to oval intraepithelial cystic spaces within the cultured bronchial epithelium that often compress adjacent epithelial cells), epithelial hyperplasia, epithelial atrophy, and/or squamous differentiation. The ciliary abnormalities caused by the mid concentration of CS were completely resolved after a 20-day recovery period. However, ciliary abnormalities persisted throughout the 20-day recovery in the high CS concentration group (Fig. 7b).

Consistent with the structural changes, CS inhibited CBF in a concentration-dependent manner after 12 repeated exposures. Cilia beating was abolished at T12, but was completely reversed after a 20-day recovery in the high CS concentration group (Fig. 7c, T12 and PT20). Examination of the expression of a ciliary structural protein, acetylated α-tubulin, revealed a similar pattern, with its expression being down-regulated after 12 repeated exposures (T12) and partially restored, but still significantly reduced relative to the clean air-exposed control, at PT20 (Fig. 7d, e).

### Induction of squamous differentiation and related changes produced by CS

**Fig. 8:**
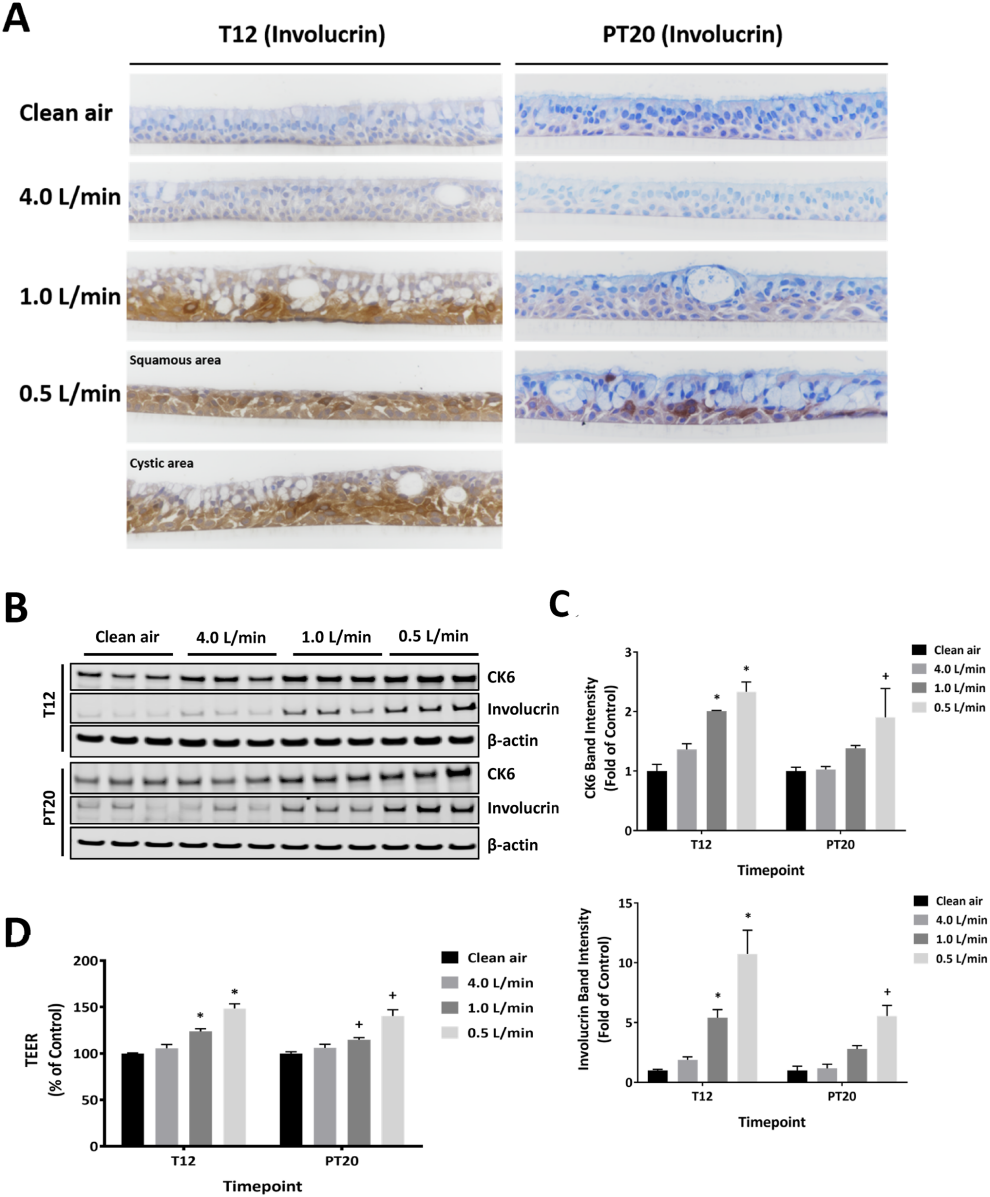
Induction of squamous differentiation by CS. The marker of squamous differentiation, involucrin, was immunohistochemically assessed at T12 and PT20 (**a**). Images were taken using 40 × magnification. Expression of two markers for squamous differentiation was measured using immunoblotting (**b**). The intensity of the bands was quantified by densitometry and normalized to the β-actin loading control (**c**). TEER was measured at the same time points (**d**). Data (*n* = 3) are expressed as means ± SEM. *^,+^*p* < 0.05 was considered statistically significant compared to the respective clean air-exposed controls by one-way ANOVA using Dunnet’s multiple comparison test (color figure online)

Repeated exposures to CS significantly increased the staining intensity for involucrin, particularly in cells with squamoid appearance (Fig. 8a, T12). Areas with squamous differentiation consisted of aggregates of polygonal- or squamous-shaped cells. A 20-day recovery partially reversed the squamous differentiation that was observed in the medium CS concentration group at the T12 sampling time (Fig. 8a, PT20). Involucrin-positive cells were still evident in cultures exposed to the high concentration of CS at the end of the recovery, suggesting that the morphological changes in this group were only partially reversed.

Consistent with the histopathological observations on squamous differentiation, the expression of both involucrin and CK6 was upregulated in a concentration-dependent manner by CS exposures (Fig. 8b, c, T12). Their expression remained elevated after a 20-day recovery, although to a lesser extent, compared to the levels measured at T12 (Fig. 8b, c, PT20), indicating partial recovery from induction of squamous differentiation after a 20-day recovery period.

A positive correlation between the extent of squamous differentiation and epithelial barrier integrity was suggested by concentration-dependent increases in TEER values following CS exposure (Fig. 8d, T12 and PT20). In addition, a reversible decrease in the number of p63-positive basal cells was observed in CS-exposed cultures (Figure S1A, T12 and PT20). The number of Ki67-positive cells, however, remained unchanged at the T12 and PT20 sampling times (Figure S1B, T12 and PT20).

### Key molecular abnormalities induced by CS

To correlate the CS-modulated biochemical and phenotypic changes to relevant molecular pathways, we performed high-throughput RNA-Seq to analyze changes in the transcriptome profiles in response to CS exposure of the ALI airway models and identified key genes involved in the proposed MOA for CS toxicity (Fig. 1b).

Significant numbers of differentially expressed genes (DEGs) were identified after 12 repeated exposures, with more genes being up-regulated than down-regulated by CS (Table 1, T12). Although the expression of most DEGs identified in the low and mid CS concentration groups returned to the baseline after a 20-day recovery, 434 genes remained differentially expressed in the high concentration group after a 20-day recovery (Table 1, PT20). Temporal transcriptome profiling revealed a total of 10,198 DEGs in the high concentration group 4 h after the first exposure (T1-4h) (Tables S1 and S2). As the number of treatments increased, the number of DEGs was found to decrease. The expression of most of the DEGs identified during the period of CS exposure (up to T12) returned to levels that were similar to the clean air control following the recovery at PT20.

**Table 1:** Number of DEGs altered by 3R4F CS at T12 and PT20

Datasets Comparison	Number of DEGs					
	T12			PT20		
	Total	Up	Down	Total	Up	Down
4.0 L/min vs. Clean air	319	173	146	0	0	0
1.0 L/min vs. Clean air	1795	1081	714	45	16	29
0.5 L/min vs. Clean air	7176	3836	3340	434	256	178

**Fig. 9:**
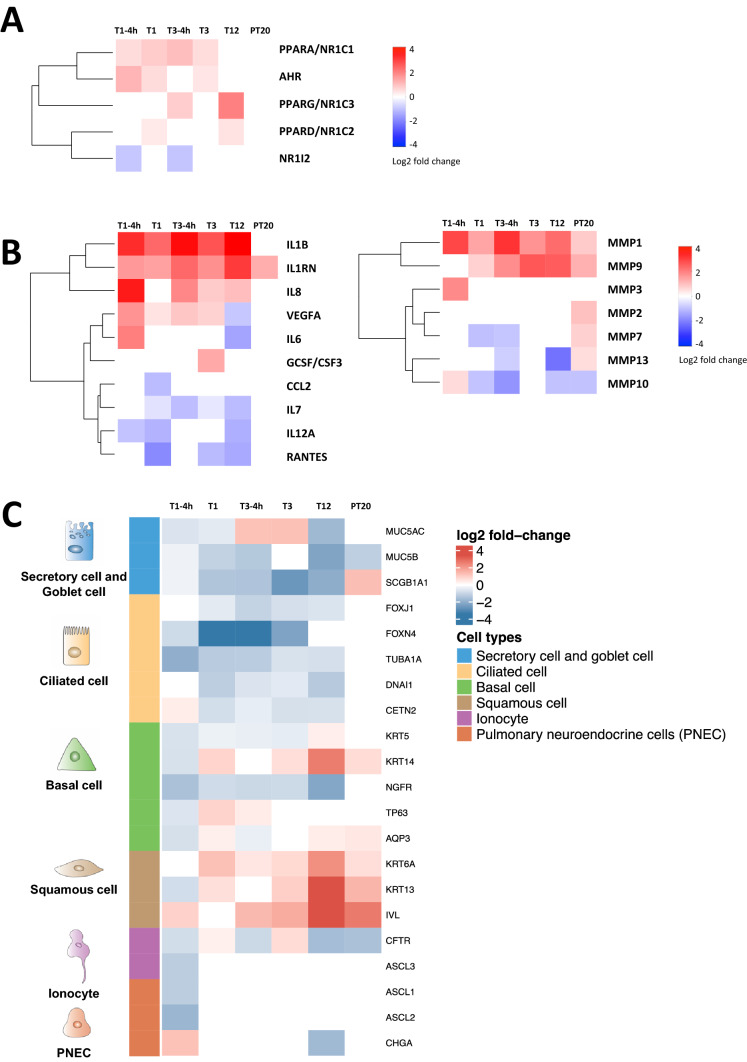
Functional analysis of DEGs modulated by CS. Time-dependent response in gene expression to CS exposure at the high concentration was analyzed. The log_2_ fold changes of the top genes (*p*_*adj*_ < .05) associated with a metabolic pathway (**a**), cytokine response (**b)**, and bronchial epithelial cell markers (**c**) are shown in the heat maps. Hierarchical clustering was used to identify sets of DEGs correlated with CS exposure at the indicated timepoints. The color scale designates the log_2_ fold changes (color figure online)

The top 20 molecular pathways (*p*_*adj*_ < 0.05) upregulated by CS exposure were identified by KEGG enrichment analysis (Figure S2). Genes involved in metabolic pathways were profoundly perturbed by both the acute and repeated CS exposures. Consistent with this observation, the expression of key transcriptional regulators of xenobiotic enzymes, such as peroxisome proliferator-activated receptors *PPARα* (*PPARA/NR1C1*) and aryl hydrocarbon receptor (*AHR*), was increased as a result of the first 3 exposures (Fig. 9a, T1-4h–T3). Upregulation of *PPARβ/δ* (*PPARD/NR1C2*) and *PPARγ* (*PPARG/NR1C3*) expression, however, required a greater number of exposures and was observed only at T12 (Fig. 9a, T12). CS transiently inhibited the expression of pregnane X receptor (*NR1/2*) 4 h after 1 or 3 exposures (Fig. 9a, T1-4h and T3-4h). Expression of these transcriptional regulators returned to the baseline level at the end of the 20-day recovery (Fig. 9a, PT20).

Differential expression of key genes involved in the proinflammatory and MMP pathways also were identified by RNA-Seq analysis. Consistent with the effects of CS on cytokine and MMP secretion (Fig. 5), the expression of *IL1RN* was upregulated by CS throughout the treatment and recovery phases (Fig. 9b). Furthermore, CS induced the expression of *IL-1β* and *IL-8*, while inhibited the expression of *VEGFA*, *IL-6*, *IL-7*, *IL-12A*, and *RANTES* (i.e., *CCL5*) after 12 repeated exposures (Fig. 9b, T12). The expression of these genes returned to the levels comparable to that of the control cultures following the recovery (Fig. 9b, PT20). The effects of CS on most *MMP* genes, however, persisted through the 20-day recovery period. In particular, the expression of *MMP-9* and *-13* was gradually modulated with an increasing number of CS exposures and culminated after 12 exposures (Fig. 9b). CS persistently upregulated the expression of *MMP-1* and *MMP-9* and downregulated the expression of *MMP-10* and *-13* throughout the treatment phase. Expression of all *MMPs*, except *MMP-3* and -*10*, remained significantly elevated at the end of the recovery (Fig. 9b, PT20).

A list of airway epithelial cell markers was identified from the literature (Choksi et al. 2014; Montoro et al. 2018; Plasschaert et al. 2018; Rock et al. 2010). Repeated CS exposure induced significant changes in the gene expression of markers for 3 major epithelial cell types, including secretory and goblet cells, ciliated cells, and basal cells. The expression of secretory and goblet cell markers, including *SCGB1A1*, *MUC5AC*, and *MUC5B*, was downregulated, while the expression of squamous cell markers, such as *KRT6A*, *KRT13*, and *IVL*, remained increased after 12 repeated CS exposures (Fig. 9c, T12). *KRT5* and *KRT14* expression was also induced at T12 (Fig. 9c, T12), indicating that a subset of the basal cells may have differentiated into squamous cells as previously reported (Montoro et al. 2018). Ciliated cell markers, including *FoxJ1*, *TUBA1A*, *DNAI1*, and *CETN2*, were all differentially expressed throughout the treatment and recovery phases (Fig. 9c), correlating well with the effects of CS on ciliary structure and function (Fig. 7). Consistently, downregulation of cilia biogenesis pathways was identified by GO enrichment analysis (Figure S3). Transient modulation was observed for the expression of ionocyte and pulmonary neuroendocrine cell (PNEC) markers, such as *ASCL3*, *ASCL1*, *ASCL2*, and *HGA*. Expression of these genes was affected only by the acute, but not the repeated CS exposures (Fig. 9c). Furthermore, the expression of *CFTR* was found to be persistently downregulated at both the T12 and PT20 sampling times, indicating the possible irreversible effects of CS on the functions of ion channels.

### Modulation of COPD-associated genes by CS

The molecular and structural abnormalities induced by CS in ALI airway cultures, including oxidative stress, altered secretion of cytokines and MMPs, and squamous differentiation, exhibit striking similarities to the key events involved in the pathogenesis of COPD (Agustí and Hogg 2019; Kirkham and Barnes 2013; Puchelle et al. 2006). We, therefore, compiled a comprehensive list of 146 genes associated with COPD from the literature (Agustí et al. 2019; Demeo et al. 2006; Ezzie et al. 2012; Gohy et al. 2015; Ning et al. 2004; Smolonska et al. 2009; Llinas et al. 2011; Morrow et al. 2019; Pierrou et al. 2007; Wadhwa et al. 2019; Steiling et al. 2009; Siafakas and Tzortzaki 2002; Sidhaye et al. 2019; Silverman 2006) and examined the pattern of their expression in response to CS exposure in the ALI cultures (Table S3).

**Fig. 10:**
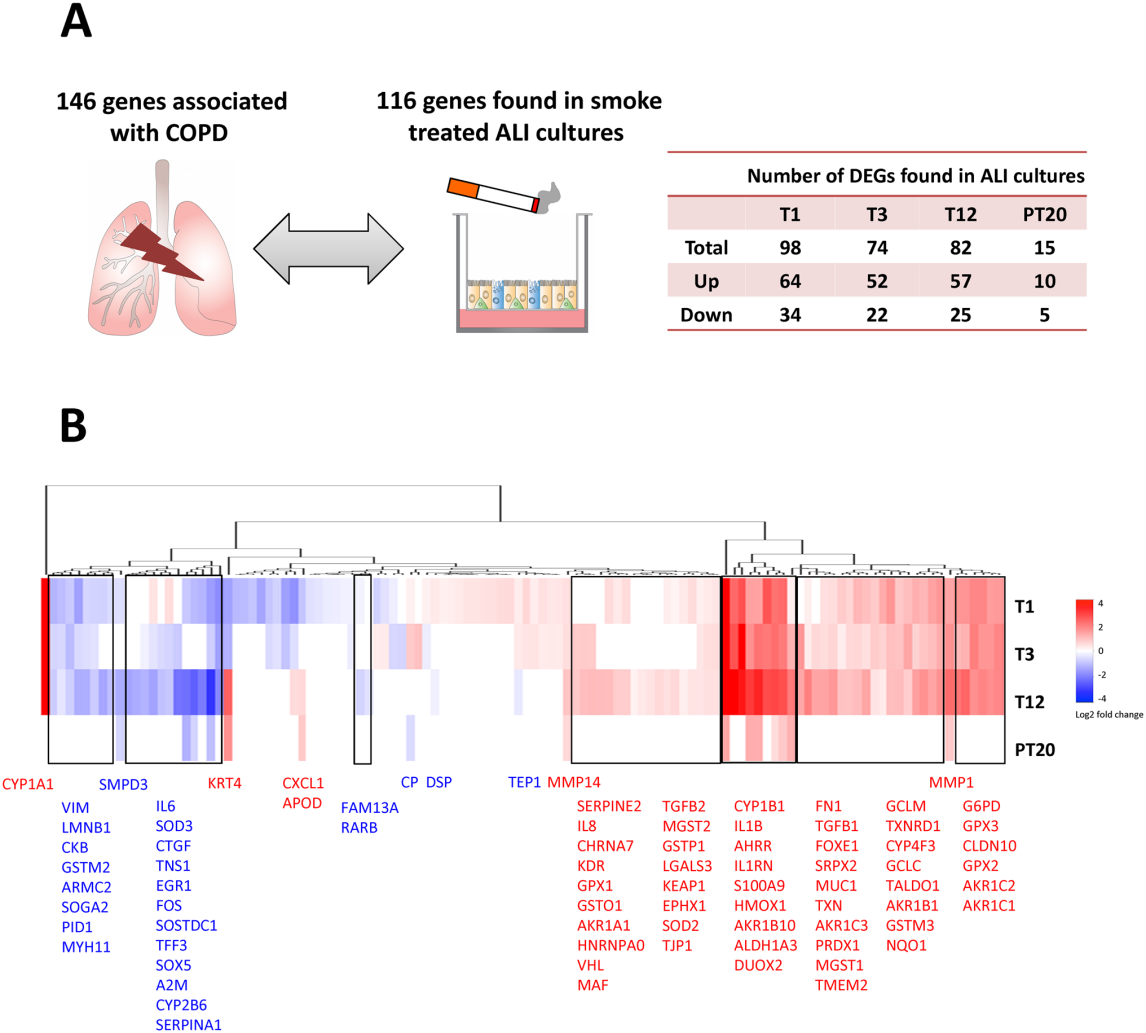
Modulation of COPD-associated genes by CS. The number of the COPD-associated genes (*p*_*adj*_ < .05) modulated by CS at T1, T3, T12 as well as PT20 is summarized (**a**). Hierarchical clustering was used to identify clusters of DEGs based on their log_2_ fold changes at the same time points (**b**). The color scale designates log_2_ fold changes. Note that DEGs with significant changes at T12 and PT20 are highlighted in red (upregulated) or blue (downregulated) in Fig. 10b (color figure online)

A total of 82 COPD-associated genes were differentially expressed in CS-exposed cultures after 12 exposures; expression of 15 genes remained differentially expressed at the end of the recovery (Fig. 10a). Clustering analysis identified clusters for a total of 116 COPD-associated genes at all time points assessed in this study (Fig. 10b, for details see Table S3). Genes involved in oxidative stress (*n* = 25), i.e., *AHRR*, *APOD*, *ALDH1A3*, *AKR1C1*, *AKR1C2*, *AKR1C3*, *AKR1B1*, *AKR1B10*, *CYP1A1*, *CYP1B1*, *CYP4F3, DUOX2, GPX2*, *GPX3*, *G6PD*, *GCLC*, *GCLM*, *GSTM3*, *HMOX1*, *MGST1*, *NQO1*, *PRDX1*, *TALDO1*, *TXN*, and *TXNRD1*, were significantly upregulated throughout the treatment phase (highlighted in red in Fig. 10b). Persistent downregulation of the growth factor/receptor genes, i.e., *RARB*, *CTGF*, *EGR1*, and *VIM,* and upregulation of inflammatory genes, i.e., *IL1B*, *IL1RN*, *IL-8*, and *CX3CL1*, was observed after 12 repeated exposures (highlighted in blue and red, respectively, in Fig. 10b).

CS-induced changes also included the upregulation of MMP genes (i.e., *MMP1* and *MMP14*) as well as genes involved in airway remodeling (i.e., *KRT4*, *CLDN10*, *FN1*, *TJP1*, *TGFB1*, and *TGFB2)*. It is noteworthy that the expression of *SERPINA1*, the deficiency and mutation of which is highly associated with the development of COPD (Brode et al. 2012; Silverman 2006; Wadhwa et al. 2019), was persistently downregulated by CS throughout the treatment and recovery phases.

## Discussion

It is anticipated that modern toxicological assessments will benefit from the development of MOA-based in vitro alternatives for safety assessment (Clippinger et al. 2018). In this study, we integrated transcriptome analysis with pathophysiological measurements and conducted a comprehensive evaluation of the toxicity responses produced by whole CS in an in vitro human ALI airway tissue model. A repeated exposure regimen was employed to examine subacute responses to CS exposure. Our findings demonstrate that CS induces a series of structural, functional, and molecular alterations in these cultures, including oxidative stress, release of proinflammatory cytokines and MMPs, impaired ciliary functionality, dysregulated expression and secretion of mucins, and squamous differentiation, that are highly consistent with the abnormalities observed in airways of smokers (Bernard et al. 1992; Herfs et al. 2012; Kirkham and Barnes 2013; Leopold et al. 2009). RNA-seq analysis further revealed a panel of DEGs that are associated with the key events in the MOA of CS toxicity. In particular, repeated CS exposures profoundly modulated the expression of genes involved in the pathogenesis of COPD, suggesting their potential as biomarkers of harm for monitoring the adverse health effects caused by CS.

Smokers are exposed to high concentrations of free radicals, reactive oxidants, and heavy metals (e.g., cadmium is known to induce oxidative stress via replacing redox-active metals, such as Fe, or inactivating antioxidant enzymes by binding to cysteine residues) (Counts et al. 2005; FDA 2012; Pappas et al. 2014; Eiserich et al. 2005; Morrow et al. 1995; Reznick et al. 1992; Nagai et al. 2006; Barreiro et al. 2013), resulting in direct or indirect oxidation of biomolecules and disturbance of the redox balance in the airways, blood, and bronchoalveolar lavage (BAL) fluid. CS-mediated oxidative stress is believed to be the key molecular initiating event underlying the pathogenesis of smoking-associated respiratory diseases, such as COPD and lung cancer (Boukhenouna et al. 2018; Gould et al. 2011). Our data demonstrated an immediate, but transient, induction of protein carbonyl formation in the ALI cultures following the first exposure. The lack of accumulation of protein carbonyls after repeated CS exposures indicates either efficient degradation by proteasomes or decomposition via retro-Michael addition of oxidized proteins (Davies 2001; Dalle-Donne et al. 2017). Disruption of glutathione homeostasis, on the other hand, exhibited temporal responses. A rapid depletion of GSH, which resulted in a concurrent decrease in GSSG formation, was observed immediately after the first exposure to CS. Following a 24-h recovery, GSH adaptive responses, such as upregulation of the rate-limiting enzymes involved in de novo GSH synthesis, may have been initiated, resulting in significant increases in both GSH and GSSG levels (Cantin 2010). By the end of the twelfth exposure, the GSH redox balance appeared to reach a new steady state, as the GSH/GSSG ratio was no longer unaffected by CS. However, the expression of HMOX-1 remained elevated at this timepoint, indicating that oxidative stress persisted throughout the CS treatment phase.

It has been reported that disruption of the redox balance by CS leads to the production of proinflammatory cytokines, i.e., IL-1β, IL-6, IL-8, and TNFα in smokers, regulation of which is closely associated with the progression of respiratory diseases, such as COPD (Zuo et al. 2014; Strzelak et al. 2018). Increased secretion of these cytokines is associated with tracheobronchial squamous metaplasia in smokers (Barnes 2004). Consistent with observations reported in smokers, the gene expression and secretion of proinflammatory cytokines (e.g., IL-1β, IL-6, and IL-8) as well as squamous differentiation were both induced in CS-exposed ALI cultures after 12 repeated exposures. Repeated CS exposure also resulted in the release of elevated amounts of G-CSF and RANTES, both of which are implicated in neutrophilic inflammatory responses and the exacerbation of neutrophilic asthma (Kim et al. 2019; Pan et al. 2000). Although higher levels of MCP-1 are reported in the bronchiolar epithelium of subjects with COPD (de Boer et al. 2000), we observed a decrease in MCP-1 secretion. Such a departure from the in vivo findings suggest limitations of this in vitro model that may be due to either the lack of other cell types, such as fibroblasts and immune cells, or an insufficient treatment duration for the cultures to acquire the full-spectrum of the phenotypes characteristic of COPD. Although airway ALI model co-cultures containing fibroblasts and immune cells are available, the addition of other cell types, such as macrophages, may introduce more variations, their suitability for toxicity testing, therefore, remains to be evaluated.

In addition to cytokines, MMPs also are involved in the recruitment of immune cells and in airway remodeling in smokers with COPD (Puchelle et al. 2006; Bergeron et al. 2009). Secretion of all the MMPs examined in this study, except MMP-9 and MMP-12, was downregulated at the end of the subacute exposure. Their secretion either returned to the baseline levels (i.e., MMP-1, -3, -7, and -13) or rather increased at the end of the recovery phase (i.e., MMP-2, -9, and -12). Compared to its effects on cytokine and chemokine secretion, CS persistently modulates MMP secretion, suggesting their longer-term, direct involvement in airway remodeling of the ALI cultures. Indeed, CS-induced increases in several MMPs, predominantly MMP-1, -2, -9 and -12, have been associated with the development and exacerbation of COPD (Barnes 2004; Churg et al. 2012).

Ciliated cells are an integral component of the mucociliary clearance apparatus that facilitates the effective removal of the endogenous mucus as well as inhaled substances (Tilley et al. 2015). Several lung diseases, including COPD and lung cancer, are characterized by abnormalities in mucociliary clearance associated with disruption of ciliary structure and function (Yaghi et al. 2012; Yaghi and Dolovich 2016). Shorter or missing cilia, compound cilia, or reduced beating frequencies are observed in smokers and likely contribute to the pathogenesis of smoking-associated lung diseases (Leopold et al. 2009; Tilley et al. 2015). Herein, we demonstrated reversible decreases in CBF by repeated CS exposure of ALI cultures. We observed not only reductions in the number and length of cilia in CS-exposed cultures but also the loss of ciliated cells in areas of the cultures undergoing epithelial remodeling. These observations were consistent with the downregulation of acetylated α-tubulin protein expression. Transcriptome profiling provided evidence for the inhibition of additional ciliary proteins by as little as one CS exposure, suggesting the potential use of these early markers for predicting ciliary damage caused by inhaled substances. In particular, the decrease in the expression of FoxJ1, which has been shown to play a key role in the differentiation of ciliated cells in a mouse ALI airway model (You et al. 2004), suggests possible impairment of ciliogenesis by CS exposures. The severity of ciliary damage (e.g., reduction in ciliary beating and shortened cilia) was positively correlated with CS concentration, with abnormalities elicited by the high concentration being only partially reversed by a 20-day recovery.

Cigarette smoking often results in exposure to high levels of polycyclic aromatic hydrocarbons (PAHs), a major risk factor for the development of lung cancer (DeMarini 2004; IARC 2004). PAHs, such as benzo[a]pyrene, are bio-activated by phase I drug metabolism enzymes, i.e., CYP1A1/2 and CYP1B1, leading to the formation of radical cations, diol-epoxides, and ortho-quinones. These reactive metabolites can react with DNA and trigger tumorigenesis (Moorthy et al. 2015). PAHs are also capable of inducing CYP1A1/2 and CYP1B1 via aryl hydrocarbon receptor (AHR)-dependent pathway that, in turn, can exacerbate the toxicity and carcinogenicity of PAHs (Omiecinski et al. 2011). In this study, we observed that CS exposure resulted in a transient induction of *AHR* (Fig. 9a, T1 and T3), accompanied by persistent upregulation of *CYP1A1* and *CYP1B1*. These findings are consistent with those reported in the airways of COPD patients and highlight the potential role of CYP1A and CYP1B family enzymes in CS-induced toxicity and carcinogenicity (Pierrou et al. 2007). In contrast to the transient upregulation of *AHR*, the expression of two isoforms of the metabolic regulator *PPAR*, i.e., *PPARβ/δ* (*PPARD/NR1C2*) and *PPARΥ* (*PPARG/NR1C3*), was induced after 12 repeated CS exposures. Recent studies in animals have uncovered a novel function for PPARs in inhibiting proinflammatory signaling pathways via the inflammatory transcription factors, such as NF-κB (Liu et al. 2017; Solleti et al. 2015). Thus, the downregulation of NF-κB-dependent genes, including *VEGFA, IL6, IL7, IL12A*, and *CCL5* (Fig. 9b, T12), that was observed in CS-exposed cultures may have resulted from the activation of PPARs.

Upregulation of genes in the aldo–keto reductase family, in particular *AKR1B1* and *AKR1B10*, may reflect a cellular defense mechanism against CS toxicity. Both AKR1B1 and AKR1B10 are capable of metabolizing toxic carbonyls, such as formaldehyde and acrolein, to less toxic metabolites, and, therefore, attenuate the electrophilic injury from CS (Barski et al. 2008). Moreover, AKR1B10 has been considered a potential biomarker of CS-associated lung cancer (Wang et al. 2010). Our data demonstrate a persistent and irreversible effect of CS on both the gene and protein expression of AKR1B10 (Fig. 10b and Figure S4), suggesting AKR1B10 may be a useful biomarker for CS exposure. It is uncertain whether or not significant changes in the expression of Phase II genes (e.g., *AKRs, ALDHs, HMOX-1*, and *NQO1*) are induced via Nrf2 pathway, which is usually activated by nucleus translocation but not necessarily by increased expression induced by the electrophiles and oxidants in CS (Müller and Hengstermann 2012).

Understanding the cellular phenotypes that are affected by CS help to identify the molecular targets responsible for the lung diseases associated with smoking (Crystal et al. 2008). Repeated exposure to CS elicited cumulative effects on the expression of a panel of genes, with their expression being most significantly altered after 12 exposures (Table S3). DO enrichment analysis of the transcriptome profiles for CS-exposed ALI cultures indicated that an increased number of lung disease-associated pathways was found after the recovery phase (Table S4, *n* = 7 at T12 vs. *n* = 12 at PT20). The predicted lung diseases include lung carcinoma, non-small cell lung carcinoma, COPD, lung adenocarcinoma, small cell lung carcinoma, pulmonary emphysema, interstitial lung disease, lung squamous cell carcinoma, and pulmonary fibrosis. These profiles reflected alteration in the expression of genes involved in extracellular matrix remodeling (i.e., *MMP1, MMP2, MMP7, MMP9, MMP13*, and *MMP14*), mucin-producing mechanisms (i.e. *SCGB1A1*), xenobiotic metabolism (i.e., *AKR1B10, CYP1B1, DUOX2, IDO1, PTGS1*, and *PTGS2*), and inflammation (i.e., *ICAM1* and *IL1RN*). Consistent with previous findings, the DO enrichment analysis conducted in this study identified the common molecular pathways underlying the pathogenesis of both COPD and lung cancer (Young and Hopkins 2011). For instance, the squamous differentiation observed in CS-exposed ALI cultures is found in the airways of smokers with COPD and squamous cell carcinoma (Bozinovski et al. 2015; Greenberg et al. 2002; Rigden et al. 2016).

CS-induced phenotypic changes, such as damage to ciliated cells and goblet cells and squamous differentiation, could be reversed to a great extent by a 20-day recovery, suggesting that airway epithelial cells have strong capacities for self-renewal and transdifferentiation. The gene expression of markers for different cell types revealed a negative correlation between the expression of squamous cell markers (i.e., *KRT6A, KRT13* and *IVL*) and ciliated (i.e., *FOXJ1, TUBA1A, DNAI* and *CETN2*)/basal cell markers (i.e., *NGFR*). Squamous cells observed in the CS-exposed cultures may be derived from ciliated cells or a subset of basal cells as previously reported in the bronchiolar epithelium of a transgenic mouse model (Park et al. 2006). Expression of the secretory cell markers, *MUC5AC, MUC5B*, and *SCGB1A1*, exhibited differential responses, with *MUC5B* and *SCGB1A1* being persistently downregulated and *MUC5AC* being fully restored at the end of the recovery period. These observations demonstrate the dynamic and differential responses of subsets of secretory cells to CS exposure. It is also possible that cell transdifferentiation may have been triggered as a rapid repair mechanism to preserve the integrity of airway epithelium (Crystal 2014; Jetten 1989; Randell 2006).

There are several limitations of our study including limited analysis of chemicals in mainstream CS. In this study, we employed QCM to quantitate the particle phase and nicotine measurement in the exposure medium as indirect indicators of smoke dosimetry. Our data on these two measurements demonstrated the linearity, even distribution, and reproducibility of CS generation throughout the exposure phase and were correlated with CS-induced functional and morphological changes observed in ALI cultures. Quantification of key CS constituents may provide more robust dosimetric parameters that are useful for correlating the in vitro and in vivo exposure doses. For instance, dilution of CS may cause the migration of semi-volatile chemical species in the particle phase to the gas phase (Alderman and Ingebrethsen 2011 and Ingbrethesen et al. 2012), resulting in higher levels of certain gas phase constituents in diluted CS. Therefore, the effects of semi-volatile CS constituents may be different between the low and high concentration groups. Recently, studies have demonstrated heterogeneous deposition of particles within the Vitrocell^®^ exposure system (Oldham et al. 2020a,b). Therefore, caution needs to be exercised when interpreting the extent of CS-induced alterations in the ALI cultures, as the biological effects are correlated with not only the concentration but also the deposition of CS aerosols (particles as well as gas phases) across the ALI cultures. A thorough investigation of the homogeneity of CS aerosol deposition within the exposure module is warranted.

It is also worth mentioning that mucus clumps were removed from the ALI cultures by a brief washing with DPBS before each CS exposure. While this may not be comparable to mucociliary clearance that occurs in vivo, our preliminary studies indicate that accumulation of mucus clumps on the apical surface of ALI cultures could significantly affect chemical uptake and absorption (data not shown). Thus, as a routine practice in our laboratory to improve the reproducibility of the assays, ALI cultures were washed with DPBS prior to the exposures.

Finally, the relevance of the in vitro CS exposure regimen employed in this study to the CS exposure experienced by habitual smokers is not assessed. It is important to note that reliable translation of the in vitro exposure scenario to the human exposure condition(s) requires extensive in-vitro to in-vivo extrapolation, which relies on thorough chemical analysis of a greater spectra of CS constituents, computational fluid dynamic-based aerosol dosimetry assessment, and physiologically-based pharmacokinetic modeling.

In summary, we have developed a repeated treatment protocol to evaluate the subacute responses to whole CS exposure in a human airway tissue model. Using an integrative approach consisting of transcriptomic analysis and pathophysiological assessments, the MOA for CS toxicity in vivo was largely replicated in this in vitro model. This study represents one of the first comprehensive analyses of the temporal progression of the key molecular and structural abnormalities induced by CS in differentiated airway ALI cultures. Furthermore, the transcriptomic analysis conducted in parallel with the assessment of physiological responses revealed key molecular targets that potentially could serve as biomarkers of harm for CS exposure.

## Supplementary Information

Below is the link to the electronic supplementary material.

Supplementary file1 (DOCX 4774 KB)

Supplementary file2 (DOCX 14 KB)

Supplementary file3 (XLSX 10083 KB)

Supplementary file4 (XLSX 28 KB)

Supplementary file5 (XLSX 15 KB)
