# Supplementary material for: Integration of transcriptome analysis with pathophysiological endpoints to evaluate cigarette smoke toxicity in an in vitro human airway tissue model
Source: Arch Toxicol. 2021 Mar 3;95(5):1739–61. doi: 10.1007/s00204-021-03008-0 (PMC8113308; doi:10.1007/s00204-021-03008-0)
Supplement: Supplementary file 1 — Supplementary file1 (DOCX 4774 KB) [file 204_2021_3008_MOESM1_ESM.docx]

**
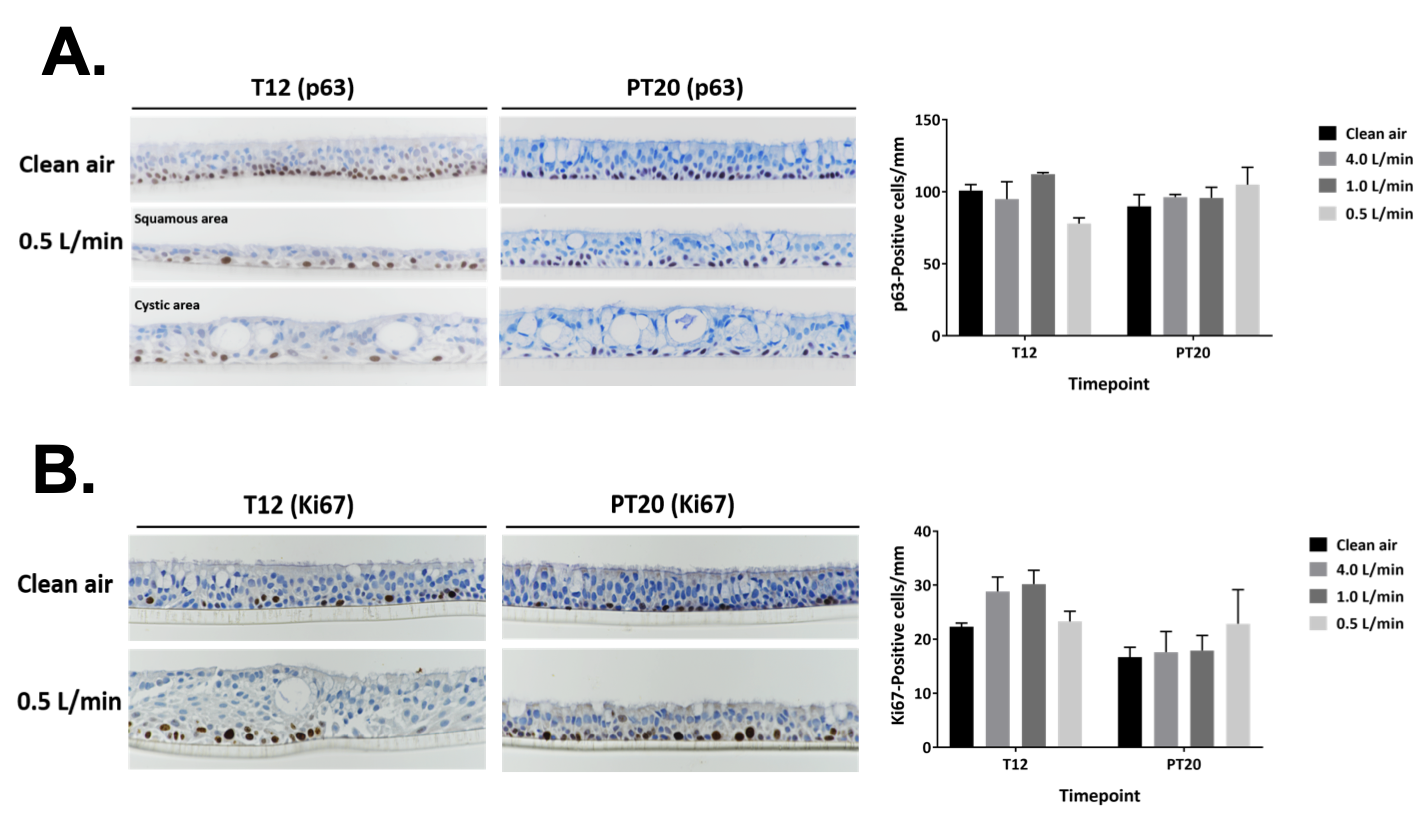
**

**Figure S1. Effects of repeated CS exposure on p63- and Ki67- expressing cells in the ALI cultures.** Histological staining of p63-positive basal cells and Ki67-positive proliferating cells in the ALI cultures following 12 exposures to the high concentration of CS (T12) as well as a 20-day recovery (PT20). A representative section of ALI cultures stained for p63 (A) and Ki67 (B) are shown on the left. Images were taken using 40*×* magnification. The number of p63- or Ki67-positive nuclei was counted over a 5-mm length of section and is shown on the right. Data (n=3) are expressed as means ± SEM. Note that a mild reduction of p63-positive basal cells was observed in cultures exposed to the high concentration of CS at T12. After a 20-day recovery, no significant changes in p63- or Ki67-positive cells were observed.


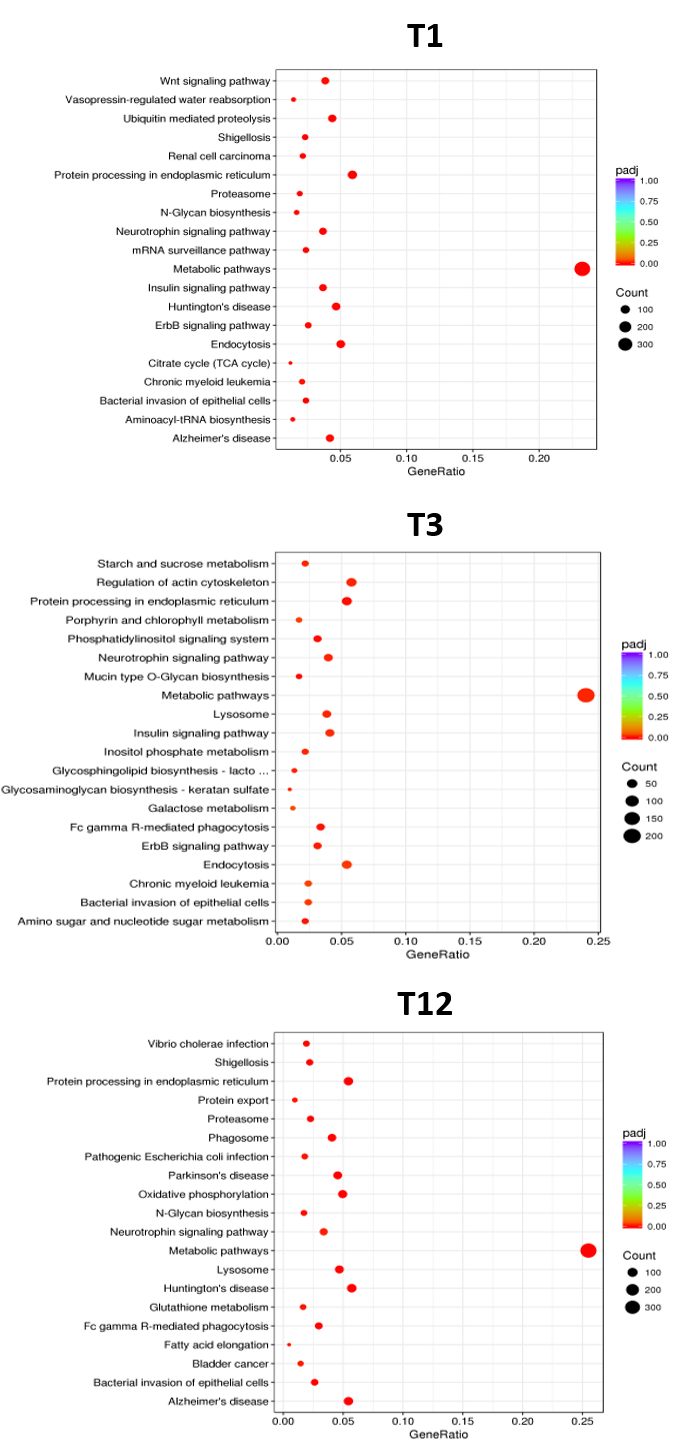


**Figure S2. Key molecular pathways modulated by CS exposure.** KEGG enrichment analysis identified the top 20 pathways (*p_adj_*<.05) that were upregulated at T1, T3, and T12. The color scale designates adjusted *p*-values. The size of the circle represents the number of differentially expressed genes (DEGs). Note that genes involved in metabolic pathways are markedly perturbed by repeated CS exposure.


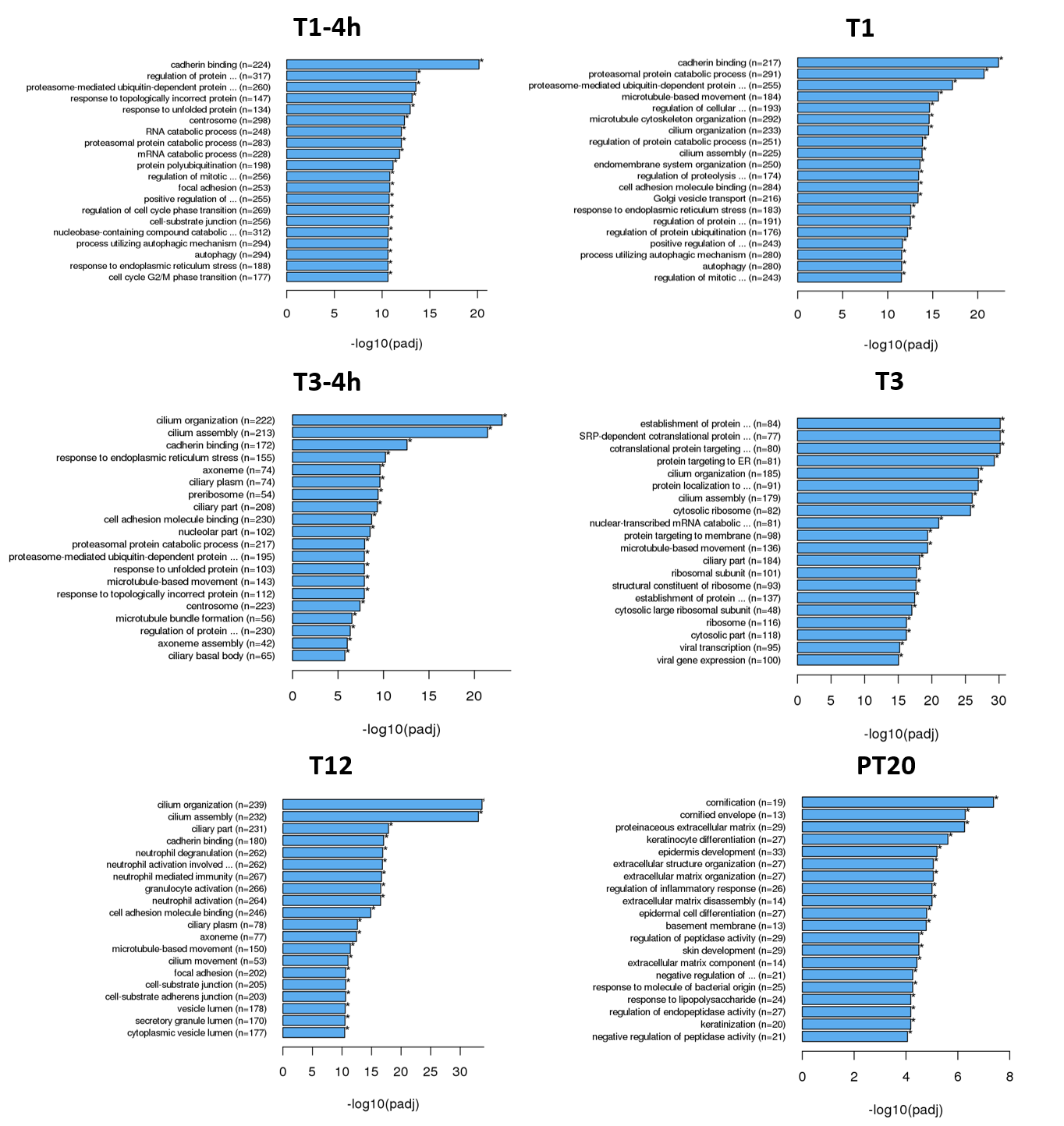


**Figure S3. Gene ontology pathway analysis of genes modulated by CS exposure.** Time-dependent responses in gene expression to CS exposure at the high concentration were analyzed. The histogram of GO enrichment analysis shows the top 20 biological networks and subnetworks (*p_adj_*<.05) altered by CS exposure at the indicated time points. Note that pathways for protein handling systems (proteasome, autophagy, and ER stress response) were affected after 1 and 3 CS exposures, while cilia biogenesis and function pathways were significantly perturbed after 12 CS exposure. Pathways, such as cornification, cornified envelope, and keratinocyte differentiation, were only identified during the recovery phase, suggesting sustained effects of CS on tissue remodeling.

**A.**


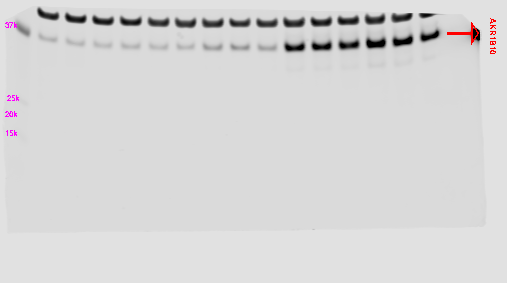

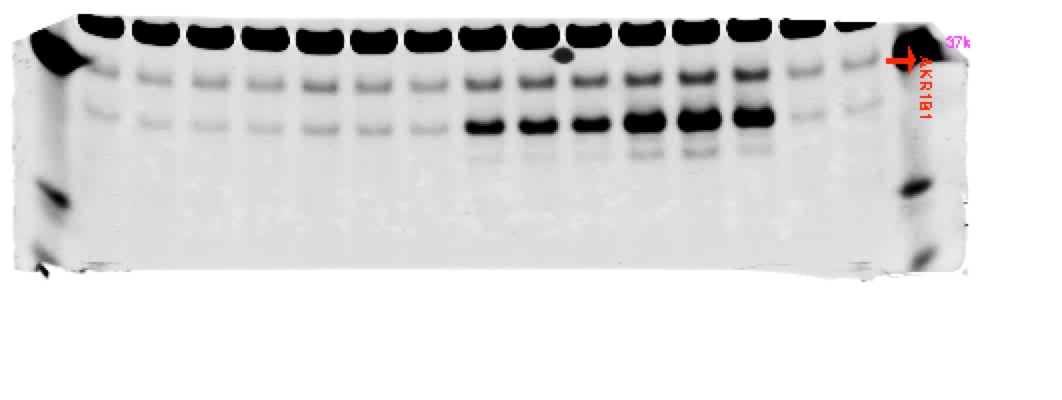

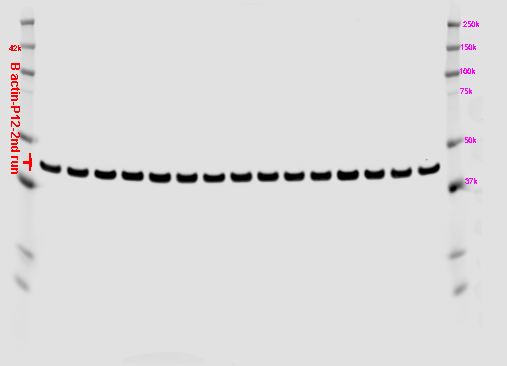


**T12**

**AKR1B10**

**AKR1B1**

**β-actin**


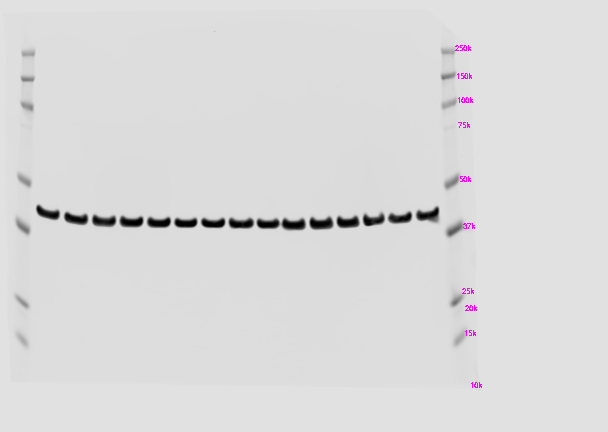

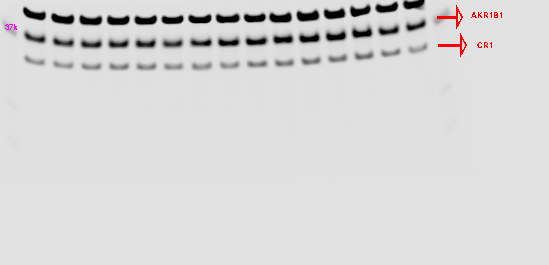

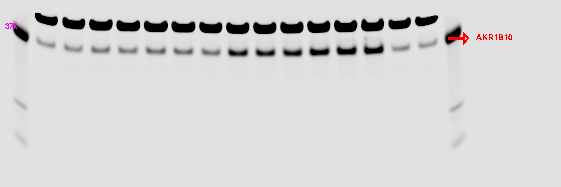


**PT20**

**AKR1B10**

**AKR1B1**

**β-actin**

**4.0 L/min**

**Clean air**

**1.0 L/min**

**0.5 L/min**

**B.**


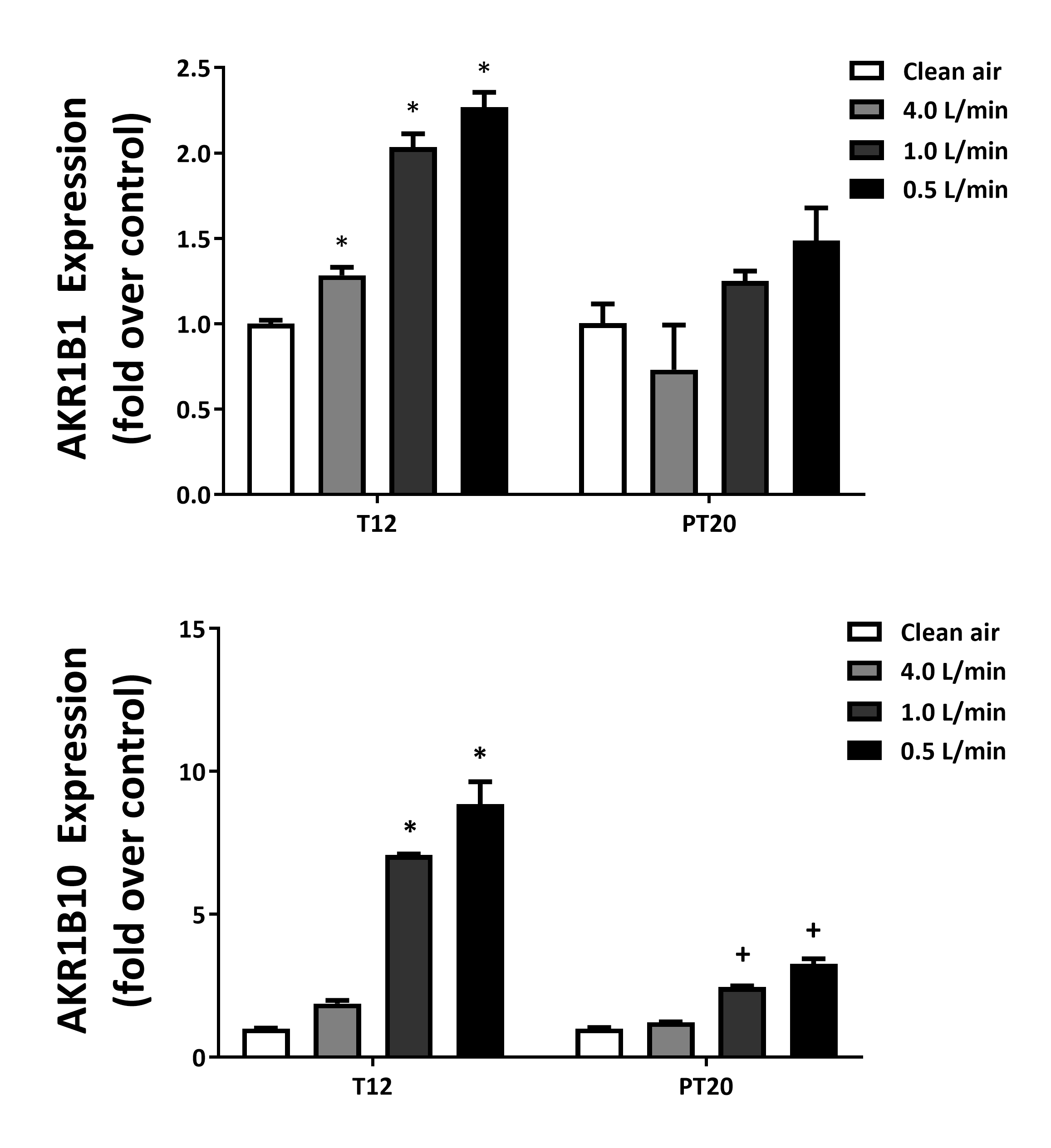


**Figure S4. Sustained induction of AKR1B1 and AKR1B10 protein expression by repeated CS exposure.** Protein expression of AKR1B1 and AKR1B10 was evaluated 24 h after 12 exposures (T12) as well as after a 20-day recovery (PT20) by immunoblotting. β-Actin was used as the loading control. Representative immunoblots are presented (A). Band intensities were quantified by densitometry (B). Data (n=3) are expressed as means ± SEM. ^*, +^*p* <.05 was considered statistically significant compared to the respective clean air-exposed controls.
