# Supplementary material for: Integration of transcriptome analysis with pathophysiological endpoints to evaluate cigarette smoke toxicity in an in vitro human airway tissue model
Source: Arch Toxicol. 2021 Mar 3;95(5):1739–61. doi: 10.1007/s00204-021-03008-0 (PMC8113308; doi:10.1007/s00204-021-03008-0)
Supplement: Supplementary file 2 — Supplementary file2 (DOCX 14 KB) [file 204_2021_3008_MOESM2_ESM.docx]

**Supplementary Table 1. Number of differentially expressed genes (DEGs) induced by 3R4F at 0.5L/min**

| **Datasets Compared** | **Timepoint** | **Number of DEGs** | | |
| --- | --- | --- | --- | --- |
|  |  | **Total** | **Up** | **Down** |
| **0.5 L/min vs Clean air** | **T1-4h** | **10198** | **5551** | **4867** |
|  | **T1** | **9068** | **5051** | **4017** |
|  | **T3-4h** | **7598** | **4340** | **3258** |
|  | **T3** | **5090** | **2506** | **2584** |
|  | **T12** | **7176** | **3836** | **3340** |
|  | **PT20** | **434** | **256** | **178** |
